# Structure-Function Studies of the Bacillus subtilis Ric Proteins Identify the Fe-S Cluster-Ligating Residues and Their Roles in Development and RNA Processing

**DOI:** 10.1128/mBio.01841-19

**Published:** 2019-09-17

**Authors:** Felix Adusei-Danso, Faisal Tarique Khaja, Micaela DeSantis, Philip D. Jeffrey, Eugenie Dubnau, Borries Demeler, Matthew B. Neiditch, David Dubnau

**Affiliations:** a Department of Microbiology, Biochemistry and Molecular Genetics, New Jersey Medical School, Rutgers University, Newark, New Jersey, USA; b Public Health Research Center of New Jersey Medical School, Newark, New Jersey, USA; c Department of Molecular Biology, Princeton University, Princeton, New Jersey, USA; d Department of Chemistry & Biochemistry, The University of Lethbridge, Alberta, Canada; University of Minnesota Medical School

**Keywords:** Ric proteins, iron sulfur cluster, *Bacillus subtilis*, RNA processing, bacterial development

## Abstract

In Bacillus subtilis, the RicA (YmcA), RicF (YlbF), and RicT (YaaT) proteins accelerate the phosphorylation of the transcription factor Spo0A, contributing to genetic competence, sporulation, and biofilm formation, and are also essential for the correct maturation of several protein-encoding and riboswitch RNAs. These proteins form a stable complex (RicAFT) that carries two [4Fe-4S]^+2^ clusters. We show here that the complex is a 1:1:1 heterotrimer, and we present the X-ray crystal structures of a RicAF heterotetramer and of a RicA dimer. We also demonstrate that one of the Fe-S clusters (cluster 1) is ligated by cysteine residues donated exclusively by RicT and can be retained when the RicT monomer is purified by itself. Cluster 2 is ligated by C167 from RicT, by C134 and C146 located near the C terminus of RicF, and by C141 at the C terminus of RicA. These findings imply the following novel arrangement: adjacent RicT residues C166 and 167 ligate clusters 1 and 2, respectively, while cluster 2 is ligated by cysteine residues from RicT, RicA, and RicF. Thus, the two clusters must lie close to one another and at the interface of the RicAFT protomers. We also show that the cluster-ligating cysteine residues, and therefore most likely both Fe-S clusters, are essential for *cggR-gapA* mRNA maturation, for the regulation of *ricF* transcript stability, and for several Ric-associated developmental phenotypes, including competence for transformation, biofilm formation, and sporulation. Finally, we present evidence that RicAFT, RicAF, and RicA and the RicT monomer may play distinct regulatory roles *in vivo*.

## Abstract (precis)

The RicA, RicF, and RicT proteins are widely conserved among the firmicute bacteria and play multiple roles in Bacillus subtilis. Among the phenotypes associated with the inactivation of these proteins are the inability to be genetically transformed or to form biofilms, a decrease in sporulation frequency, and changes in the stability and maturation of multiple RNA species. Despite their importance, the molecular mechanisms of Ric protein activities have not been elucidated and the roles of the two iron-sulfur clusters on the complex of the three proteins are not understood. To unravel the mechanisms of Ric action, molecular characterization of the complex and of its constituent proteins is essential. This report represents a major step toward understanding the structures of the Ric proteins, the arrangement and roles of the Fe-S clusters, and the phenotypes associated with Ric mutations.

## INTRODUCTION

RicA (YmcA), RicF (YlbF), and RicT (YaaT) were first identified as proteins that are individually required for a number of developmental adaptations in Bacillus subtilis, including competence for transformation, sporulation, and the formation of biofilms (1–3). Coexpressed in Escherichia coli, the three proteins were isolated in the form of a stable complex (4, 5). Coimmunoprecipitation experiments demonstrated all three pairwise interactions in B. subtilis, and bacterial 2-hybrid experiments in E. coli verified these interactions (4, 6).

Genetic and biochemical investigations provided evidence that the Ric proteins stimulate the phosphorelay that produces Spo0A-P, a transcription factor needed for the formation of spores, biofilms, and K-state cells that are competent for genetic transformation (4, 5, 7). In particular, *ric* deletions were bypassed for early spore gene expression by mutations that are known to suppress loss-of-function alleles of genes encoding phosphorelay proteins and recapitulation of the phosphorelay *in vitro* with purified proteins revealed stimulation in the presence of the RicAFT complex, suggesting that the phosphorelay effect was mediated by direct interactions. Nevertheless, genetic experiments clearly demonstrated that the Ric proteins play an important role in addition to their stimulation of the phosphorelay (7). The latter finding was consistent with reports indicating that the Ric proteins are required for the efficient maturation of certain RNA transcripts, most likely by association with the Rny nuclease (6, 8). Because the Ric proteins are encoded by firmicutes that do not express Spo0A (5), it is possible that RNA maturation is a more general function and that the Ric proteins were coopted for stimulation of the phosphorelay.

An additional important discovery was that the RicAFT complex carries two oxygen-sensitive [4Fe-4S]^+2^ clusters (5) giving rise to the name Ric (regulatory iron-sulfur complex). This finding was based on UV-visible light (UV-VIS) and Mossbauer spectroscopy and on measurements of the Fe and S content of purified complexes. To better understand the molecular mechanism by which the Ric proteins accomplish their *in vivo* functions, it is desirable to characterize them more completely with regard to structure to learn how they coordinate their clusters and how the proteins interact with one another *in vivo*.

In the present work, we purified a soluble RicT monomer, a dimer of RicA, a probable RicAF tetramer, and a RicAFT trimer. We solved the X-ray crystal structures of RicA and of RicAF. We also show that the RicAFT has 1:1:1 stoichiometry and that the RicT monomer carries a single Fe-S cluster. Evidence supports a model in which the second cluster is held at the interfaces of RicT, RicA, and RicF and is ligated by cysteine residues contributed by each of these subunits. Evidence is also presented that Ric protein forms with different compositions exist *in vivo* and that these may play distinct regulatory roles in B. subtilis. Finally, we have shown that the cysteine residues that ligate the clusters are required for their functions in sporulation and biofilm formation and are also required for the roles of the Ric proteins in RNA maturation, including the autoregulation of *ricF* transcript stability. These findings increase our understanding of Ric protein biochemistry and further suggest the involvement of these proteins in a complex regulatory system that has pleiotropic effects on the biology of B. subtilis and presumably in other firmicutes.

## RESULTS

### Isolation of Ric protein forms.

**FIG 1:**
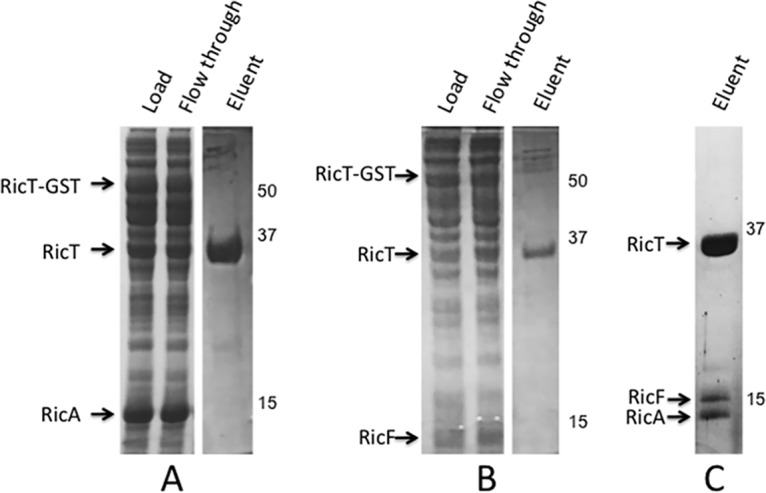
RicT-GST cannot pull down RicA in the absence of RicF (A) or RicF in the absence of RicA (B). Panel C shows the eluent after PreScission protease cleavage when all three proteins were coexpressed. The inset images show Coomassie blue-stained SD-PAGE gels.

Coexpression of the three Ric proteins in E. coli established that they interact to form a stable complex containing RicT, RicA, and RicF (4, 5). To determine if the proteins were soluble when expressed individually and to determine which pairwise complexes could be isolated in soluble form, we expressed single Ric proteins in E. coli alone and in all pairwise combinations, always with one component fused to glutathione *S*-transferase (GST). An HRV-3C protease site was placed between the GST moiety and the tagged Ric protein, permitting elution from a glutathione resin by on-column protease digestion. In addition to the complex containing all three proteins, soluble RicT and RicA and a complex of RicA and RicF were each obtained in good yield and with acceptable purity (see Fig. S1 in the supplemental material). With the GST tag placed on RicT in vectors coexpressing only RicA (Fig. 1A) or RicF (Fig. 1B), each of latter proteins was not eluted from the glutathione column by HRV-3C protease cleavage but was instead found in the flowthrough fraction. Thus, we were able to demonstrate a stable association of RicT with RicA or RicF only when all three proteins were coexpressed to form RicAFT, as shown in Fig. 1C. With GST-RicF or His-SUMO-RicF expressed by itself, most of the protein was insoluble under the conditions used, although small amounts were detected in the soluble fraction. Thus, RicT, RicA, and RicAF and a complex containing all three proteins could be isolated in good yield and in soluble form.

10.1128/mBio.01841-19.1 FIG S1 Purity of typical RicT, RicA, and RicAF preparations. The B. subtilis proteins were analyzed by SEC, and the fractions indicated within the dotted lines were analyzed by SDS-PAGE and stained with Coomassie blue, as shown in the inserts. Note that the column used for the RicA preparation differed in size from that used for RicT and RicAF. Download FIG S1, PDF file, 0.7 MB. Copyright © 2019 Adusei-Danso et al. 2019 Adusei-Danso et al. This content is distributed under the terms of the Creative Commons Attribution 4.0 International license.

### Subunit stoichiometries of RicT and RicAFT.

**FIG 2:**
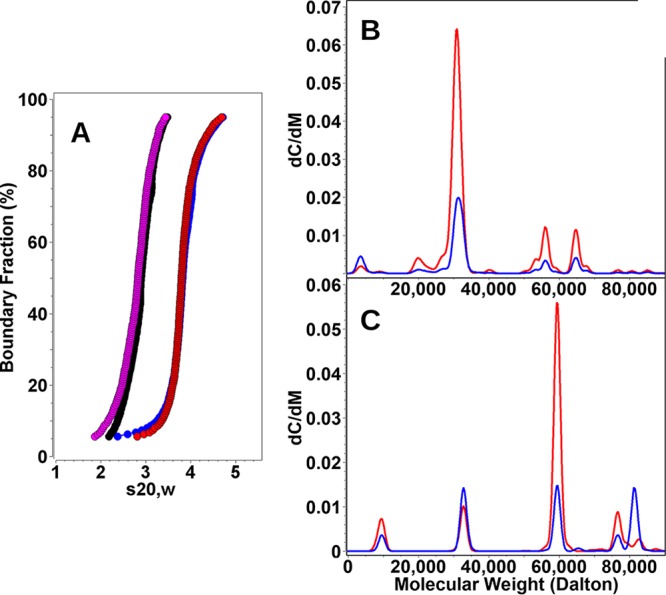
(A) Integral s-value distribution plots of RicT*^Gst^* at low (magenta) and high (black) concentrations and of (RicAFT)*^Ban^* at low (blue) and high (red) concentrations. (B and C) Molar mass distributions are shown for RicT*^Gst^* (B) and for (RicAFT)*^Ban^* (C). The red and blue lines represent 42 and 15 μM samples, respectively, for RicT (B) and 15 and 8.5 μM samples, respectively, for RicAFT (C).

To determine the stoichiometries of RicT alone and in the RicAFT complex, we used analytical ultracentrifugation (AUC). Preliminary sedimentation velocity (SV) AUC experiments showed that RicAFT from B. subtilis (RicAFT*^Bsu^*) was contaminated with nonstoichiometric RicT, and size exclusion chromatography (SEC) also provided evidence for the presence of free RicT (Fig. S2). The amount of nonstoichiometric RicT did not vary as a function of concentration in preliminary AUC experiments and therefore most likely represented production resulting from an imbalance in the levels of expression of the proteins in E. coli rather than from dissociation. This imbalance was less evident with complexes formed with Geobacillus stearothermophilus and Bacillus anthracis Ric proteins, which yielded relatively monodispersed preparations of the RicAFT complex. Examples of typical preparations are shown in Fig. S2. The sequences of the three Ric proteins from G. stearothermophilus and B. anthracis are quite similar to those of their B. subtilis orthologs (see Table S1 in the supplemental material). Because the highest purities and yields were generally obtained for the RicAFT complex from B. anthracis and for RicT from G. stearothermophilus, SV experiments and most of the subsequent biochemical work were carried out using these proteins. SV experiments characterize the solution behavior of macromolecules and report the sedimentation and diffusion behavior of all species in a mixture as well as their partial concentrations, buoyant molecular weights, and anisotropies. Results of analysis of the data are described in detail in Materials and Methods and revealed that while neither RicT of G. stearothermophilus (RicT*^Gst^*) nor RicA of B. anthracis (RicAFT*^Ban^*) was completely homogeneous, they did not change their sedimentation profiles at two different concentrations, suggesting the absence of mass action effects within the examined concentration range as shown by the integral s-value distribution plots (Fig. 2A). Molar masses estimated from the global 2-dimensional-spectrum-Monte Carlo analysis, performed using data sets from both concentrations (Fig. 2B and C), revealed major species in each case with molar masses that were in excellent agreement with the theoretical molar masses derived from the sequences and exhibiting moderate anisotropies (Fig. S3). The data suggested that RicT is monomeric (molar mass, 30.5 kDa; theoretical molar mass, 31.29 kDa) (Fig. 2B; see also Fig. S3A) and that RicAFT forms a 1:1:1 complex (molar mass, 64.7 kDa; theoretical molar mass, 65.29 kDa) (Fig. 2C; see also Fig. S3B).

10.1128/mBio.01841-19.2 FIG S2 Purification of RicAFT from B. subtilis, B. anthracis, and G. stearothermophilus. Size exclusion profiles from a S200 column are presented together with PAGE results from samples taken as indicated within the dotted lines. Download FIG S2, PDF file, 0.7 MB. Copyright © 2019 Adusei-Danso et al. 2019 Adusei-Danso et al. This content is distributed under the terms of the Creative Commons Attribution 4.0 International license.

10.1128/mBio.01841-19.3 FIG S3 Global 2-dimensional spectrum-Monte Carlo analysis for (A) RicT*Gst* and (B) (RicAFT)*^Ban^*. Anisotropies of each species found in the mixture are shown as a function of molar mass. Relative concentrations of each species are indicated by their color density; darker color indicates a higher partial concentration. Download FIG S3, PDF file, 0.2 MB. Copyright © 2019 Adusei-Danso et al. 2019 Adusei-Danso et al. This content is distributed under the terms of the Creative Commons Attribution 4.0 International license.

10.1128/mBio.01841-19.7 TABLE S1 Identities and similarities of Ric proteins. Download Table S1, PDF file, 0.03 MB. Copyright © 2019 Adusei-Danso et al. 2019 Adusei-Danso et al. This content is distributed under the terms of the Creative Commons Attribution 4.0 International license.

### X-ray crystal structures of RicA and RicAF.

**FIG 3:**
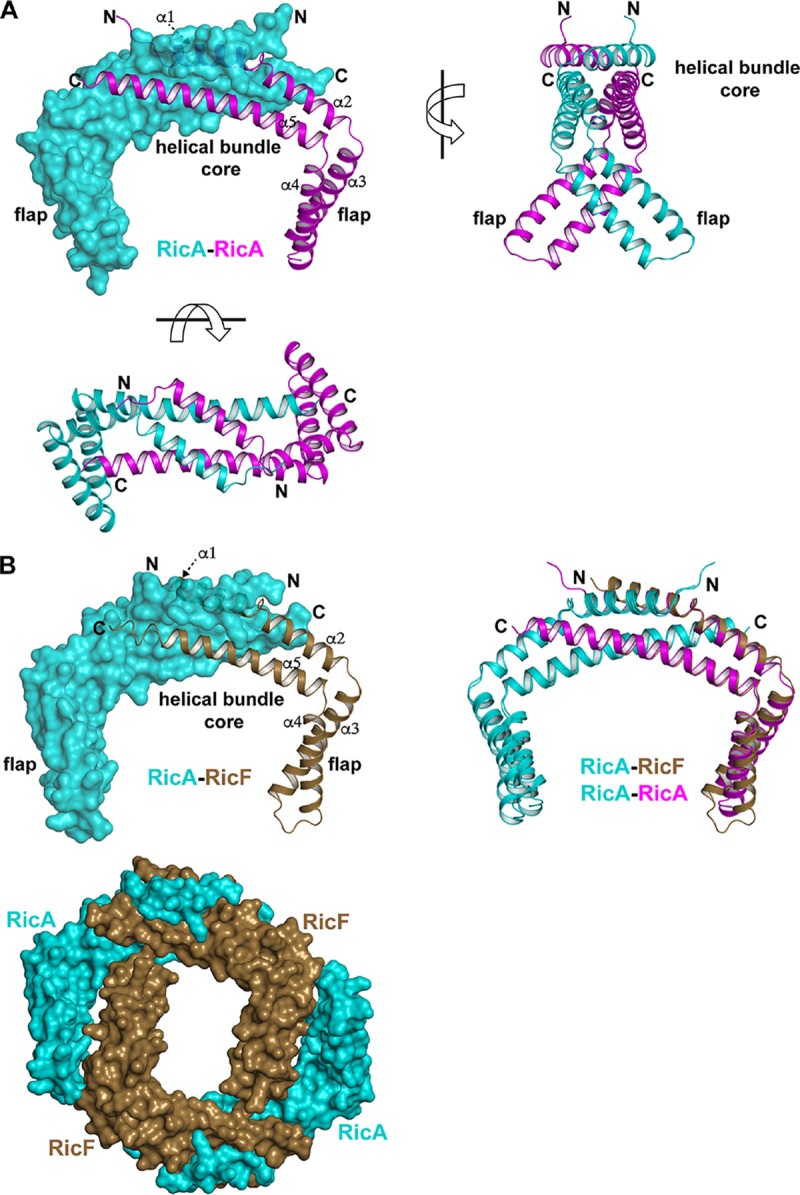
RicA and RicA:RicF crystal structures. (A) The RicA dimer viewed from the front, top, and side (top left panel, bottom left panel, and top right panel, respectively). In the front view, one RicA protomer is depicted as a transparent surface and the other as a cartoon (spirals). In the other panels, both RicA protomers are depicted as cartoons. The arrow in the front view points to the partially obscured cartoon helix α1. (B) (Top left) Front view of the RicA:RicF dimer. RicA is depicted as a transparent surface and RicF as a cartoon. The arrow points to RicF helix α1 (partially obscured). (Top right) Structural alignment of the RicA dimer and RicA:RicF. (Bottom) Front view of the RicA:RicF crystallographic tetramer. The surfaces of RicA and RicF are depicted.

To understand the molecular basis of Ric function, we are carrying out crystallographic studies of the complex and its components. So far, we have determined the structures of RicA (Fig. 3A) and RicAF (Fig. 3B). RicA and RicAF crystals were identified in high-throughput crystallization screens using purified RicAF complex with C-terminal truncations. There is one RicA protomer in the RicA crystallographic asymmetric unit, but, consistent with gel filtration results (not shown), Pisa complex analysis results (9), and the previously deposited but unpublished structure of RicA (PDB identifier [ID] 2PIH), which has a dimeric asymmetric unit, we propose that, in the absence of RicF and RicT, RicA forms the depicted homodimeric complex (Fig. 3A). The SEC profile shown in Fig. S1 is consistent with RicA existing as a dimer in solution. Each RicA protomer contains five α-helices, and dimerization is mediated by intermolecular contacts between helices α1, α2, and α5, forming a core antiparallel helical bundle that buries 1,887 Å^2^ of surface area. Outside this region, in each RicA protomer, helices α3 and α4 together form flaps that extend in a nearly orthogonal direction from the helical bundle core.

**FIG 4:**
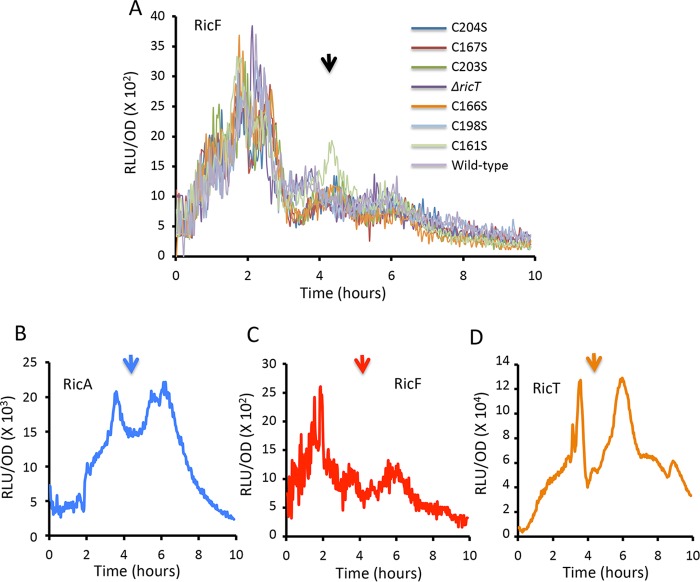
Expression from P*ricF* (A and C), P*ricA* (B), and P*ricT* (D) luciferase fusions. (A) Effects of C → S point mutations in *ricT* on expression from P*ricF*. Panels B, C, and D show results from wild-type strains growing in LB. The vertical arrows mark *T*_0_, the time of transition to the stationary phase. RLU, relative light units.

Despite the fact that RicA and RicF have only about 20% sequence identity, their monomeric structures as well as the structures of RicA and RicAF are nearly identical. In fact, superposition of RicA dimer and of RicAF revealed a root mean square deviation (RMSD) value for 166 aligned *C*_α_ of 0.982 Å (Fig. 3A; see also Fig. 4B). The most poorly aligned portions of these structures occurred in the α3-α4 flaps (Fig. 3A), suggesting that there may be some flexibility in the turns connecting α3 to α4 and α4 to α5. We note that the gap separating the RicA and RicF flaps is relatively large (∼50 Å), and we speculate that RicT may occupy this space in the RicAFT complex.

As noted above, the elution volume of RicA in SEC is consistent with it being a dimer (32.04 kDa). Despite the structural similarity of RicA and RicAF, the SEC results suggest the RicAF forms a higher-order oligomer (Fig. S1). Consistent with the RicAF SEC results, we identified a RicAF crystallographic tetramer (Fig. 3B). Thus, we tentatively conclude that RicAF is a tetramer *in vitro*, consisting of two molecules each of RicA and of RicF, while RicA is a homodimer. We have no evidence for or against the hypothesis that RicAF is a tetramer *in vivo*.

### Different Ric protein complexes may exist *in vivo*.

As noted above, several soluble forms of the Ric proteins were isolated following expression in E. coli, in addition to the RicAFT trimer, notably, the RicT monomer, RicAF, and the RicA dimer, suggesting the possibility that multiple forms may coexist in B. subtilis. To address this issue, we determined the numbers of molecules of each protein per cell by quantitative Western blotting, reasoning that if only the 1:1:1 ternary complex form existed *in vivo*, the three proteins would be present in equimolar amounts. The data in Table 1 show that this was not the case. RicT was considerably more abundant than RicA, which in turn was more abundant than RicF. Because the three proteins are not equally abundant, it is likely that that two or more associative forms exist *in vivo*, most likely the RicT monomer, the RicAFT trimer, and possibly the RicA dimer, raising the further possibility that the various forms play distinct roles. Interestingly, the abundances of the three proteins, as well as their relative proportions, in cells grown in lysogeny broth (LB) differed from those seen in cells grown in glucose minimal medium. In fact, results of analysis performed using promoter fusions to the gene for firefly luciferase (*luc*) showed distinct transcription patterns during growth for each of the three genes, which are harbored in separate transcription units (Fig. 4B to D). In accordance with the relative abundances of the proteins (Table 1), the maximum transcription rates were in the order *ricT* > *ricA* > *ricF*, suggesting that the differences in abundance are at least partly determined at the level of transcription.

**TABLE 1:** Ric protein amounts per cell

Protein	No. of molecules/cell × 10^3^^*a*^	
	LB medium	Minimal medium
RicA	22 ± 1.0	3.7 ± 1.9
RicF	6.9 ± 0.07	0.34 ± 0.25
RicT	600 ± 17	11.5 ± 6.3

a All values represent averages ± standard deviations, based on results from three biological replicates. Cells were harvested in the late log phase. Purified protein samples were included on each gel to construct standard curves. LB medium, standard lysogeny broth.

**FIG 5:**
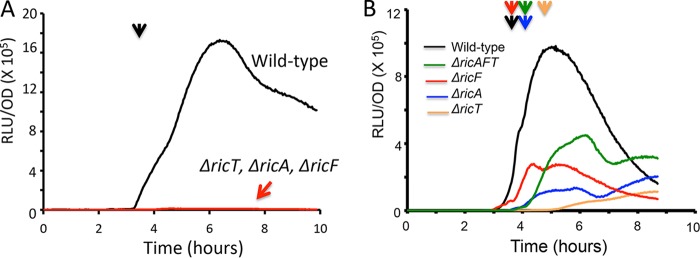
Effects of *ric* gene deletions on the expression of P*spoIIG-luc* in the domesticated (IS*75*) (A) and undomesticated (ATCC 3610) (B) backgrounds. The vertical arrows indicate the time of transition to stationary phase (*T*_0_).

If various Ric protein complexes play distinct roles, their associated phenotypes may also be distinct. To address this issue, we first turned our attention to the Spo0A-P-dependent *spoIIG* promoter, a reporter for sporulation. Our results suggest that forms containing only RicA and RicF may modulate *spoIIG* expression. For these experiments, it was instructive to compare two strain backgrounds; a commonly used B. subtilis laboratory strain and the undomesticated isolate ATCC 3610. This is because all of the domesticated strains of B. subtilis carry a partial loss-of-function point mutation in SigH, a sigma factor required for full *spoIIG* expression (10). As a result of this mutation, the expression levels of spore formation (4) and of *spoIIG* are extremely sensitive to each of the individual *ric* knockouts (Fig. 5A). In contrast, when the wild-type *sigH* is present, as in the undomesticated strain, expression of *spoIIG-luc* is affected only moderately by the individual *ric* deletions (Fig. 5B). Remarkably, simultaneous deletion of all three *ric* genes had a lesser effect than the deletion of *ricT*, suggesting that the presence of RicA and RicF in the absence of RicT might inhibit *spoIIG* expression (Fig. 5B). In fact, when *ricA* and *ricF* were simultaneously overexpressed from an inducible promoter in an otherwise wild-type domesticated strain, an inhibition of expression from the *spoIIG* promoter was consistently observed (Fig. S4). These data raise the possibility that RicA/RicF-containing forms may modulate *spoIIG* expression *in vivo*.

10.1128/mBio.01841-19.4 FIG S4 Effect of overproduction of *ricF* and *ricA* from an IPTG-inducible promoter on P*spoIIG-luc* expression. Wild-type and Δ*ricT* strains are shown for comparison. The vertical arrow marks *T*_0_, the time of transition to the stationary phase. Download FIG S4, PDF file, 0.2 MB. Copyright © 2019 Adusei-Danso et al. 2019 Adusei-Danso et al. This content is distributed under the terms of the Creative Commons Attribution 4.0 International license.

**FIG 6:**
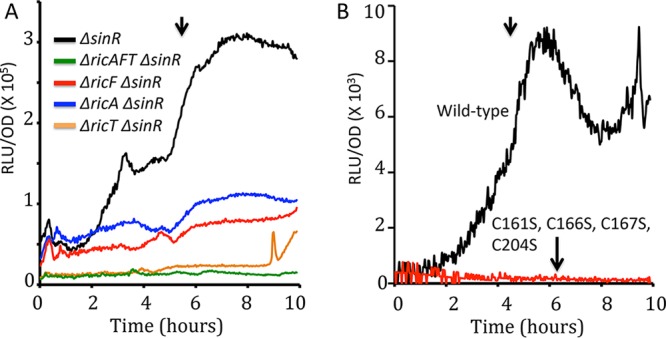
Effects of *ric* mutations on expression from a P*tapA-luc* construct in ATCC 3610. (A) All strains carried a deletion of *sinR*. (B) Effects of C → S mutations in *ricT* in strains that are wild type for *sinR*. Note the differences in scale in the backgrounds with and without *sinR*. The vertical arrows mark T_0_, the time of transition to the stationary phase.

A different result was obtained when the effects of individual deletions on P*tapA* wtested in ATCC 3610. *tapA* is an important biofilm-specific gene that is dependent on the *ric* genes for its expression (11, 12). We have presented evidence that this dependence is due in part to an effect on the phosphorelay, which is needed to counteract the effect of SinR, a potent repressor of *tapA* (5, 7, 13, 14). An alternative explanation based on the regulation of *sinR* mRNA stability has been proposed (6). However, deletion of *sinR* results in only partial bypasses of *ric* knockouts for *tapA* expression, showing that there is an additional important effect of the Ric proteins that is independent of any effect on SinR (7). To study this independent effect, we used *ric* mutations in a *ΔsinR* background. Panel A of Fig. 6 shows that, as with P*spoIIG*, the *ric* deletants exhibited different levels of P*tapA* expression. Unlike the situation with *spoIIG-luc*, the *ΔricT* and the *ΔricAFT* strains showed a stronger effect than was seen with elimination of either *ricA* or *ricF*. Although the wild-type *ΔsinR* mutant exhibited a strong dependence of *tapA* expression on the growth stage, the low-level expression in the *ric ΔsinR* mutants did not, suggesting that all three proteins are needed for regulation in response to cell density or growth stage, distinct from their effects on expression early in growth. These experiments and the results obtained with the *spoIIG* reporter are suggestive of independent roles for various Ric protein forms, although this idea needs confirmation. Importantly, the pronounced effects of the deletion mutations in a *ΔsinR* background underscore that the Ric proteins are needed for biofilm formation in addition to their mitigation of SinR activity.

### One Fe-S cluster is coordinated by RicT cysteine residues and the second at the interface of all three subunits.

RicT contains six cysteine residues that are conserved with completely consistent spacing (CX_4_CCX_30_CX_4_CC) in all the firmicutes that encode this protein. Complete alignments of RicT from B. subtilis, B. anthracis, and G. stearothermophilus are shown in Fig. S5A, and conservation of the C-rich region is shown for additional representatives of several firmicute genera in Fig. S5B. An anaerobically purified mutant (RicAFT)*^Bsu^* complex in which the six conserved RicT cysteine residues were simultaneously converted to serine yielded a completely colorless preparation (V. Carabetta, A. Tanner, and D. Dubnau, not shown), in contrast with the wild-type complex, which exhibited a deep brown color (5). Reconstitution of this mutant complex by incubation with LiS and FeCl_3_ introduced only 0.12 Fe atoms per Ric complex, showing that at least some of these conserved residues were required for ligation of both of the clusters normally carried on RicAFT.

10.1128/mBio.01841-19.5 FIG S5 Alignments of Ric proteins. (A) RicT from B. subtilis, G. stearothermophilus, and B. anthracis. (B) Alignments of a portion of RicT surrounding the conserved C residues (indicated in boldface). Sequences from representative firmicutes in addition to B. subtilis, G. stearothermophilus, and B. anthracis are shown as follows: Lmo (Listeria monocytogenes), Sau (Staphylococcus aureus), Lla (Lactococcus lactis), Spy (Streptococcus pyogenes), Hmo (Heliobacterium modesticaldum), Mce (Megasphaera cerevisiae), Cdi (Clostridioides difficile). (C) Alignment of the C-terminal tail of RicF with the conserved C residues (indicated in boldface). (D) Alignment of the C-terminal tail of RicA with terminal C residues (indicated in boldface). Download FIG S5, PDF file, 0.03 MB. Copyright © 2019 Adusei-Danso et al. 2019 Adusei-Danso et al. This content is distributed under the terms of the Creative Commons Attribution 4.0 International license.

We next determined if the RicT*^Gst^* monomer and the (RicAF)*^Bsu^* complex could carry Fe-S clusters. Isolated from E. coli under anaerobic conditions, (RicAF)*^Bsu^* exhibited no obvious color. After attempted reconstitution by incubation with FeCl_3_ and LiS, little if any Fe above the background was detected (0.23 Fe atoms/heterodimer), indicating that this complex could not accept Fe-S clusters in the absence of RicT. In contrast, the RicT*^Gst^* monomer accepted an average of 4.3 ± 0.1 mol of Fe per mole of protein, corresponding to a single [4Fe:4S] cluster/monomer (Table 2). To gain information concerning the RicT residues that coordinate this cluster, the six conserved C residues were individually changed to serine in RicT*^Gst^* and the resulting mutant proteins were expressed and purified from E. coli. Fe content was then measured for each of the mutants after reconstitution (Table 2). (In the following text, the numbering of residues in RicT, RicF, and RicA refers to those of B. subtilis [Fig. S6]). In two independent batches of anaerobically purified mutant complexes, the C161S, C166S, C198S, and C203S mutant proteins each accepted average amounts ranging from 0.5 to 1.3 Fe atoms per RicT monomer (Table 2) and their solutions appeared colorless, showing that these residues were required to form the cluster on RicT (cluster 1), most likely directly coordinating the Fe atoms in this cluster. In contrast, the C167S and C204S mutant RicT*^Gst^* proteins accepted about 4 atoms of Fe per molecule, indicating that these residues were not required to ligate its single [4Fe:4S] cluster. In fact, we know of no experimentally documented example of a protein in which vicinal cysteine residues (e.g., RicT 166/167 and 203/204) coordinate a single Fe-S cluster.

**TABLE 2:** Iron content of Ric proteins and complexes

Protein	No. of Fe atoms/ protein molecule (avg)^*a*^^,^^*b*^
RicT*^Gst^*	
Wild type	4.7, 4.1, 4.3,^*c*^ 4.2^*d*^ (**4.3**)
C161S	1.2, 1.0 (**1.1**)
C166S	0.5, 1.1 (**0.8**)
C167S	4.2, 3.8 (**4.0**)
C198S	0.6, 0.3 (**0.5**)
C203S	1.9, 0.7 (**1.3**)
C204S	4.5, 4.3 (**4.4**)

(RicAFT)*^Ban^*	
Wild type	9.0, 8.8 (**8.9**)^*e*^
RicTC161S	4.3, 4.6 (**4.5**)
RicT C167S	4.6, 3.8 (**4.2**)
RicT C204S	7.4, 8.5 (**8.0**)
RicF C144A or S^*f*^	7.2, *7.8* (**7.5**)
RicF C146A or S^*f*^	4.8, *4.3* (**4.6**)
RicF C134A or S^*f*^	4.4, 3.7, *3.7* (**3.9**)
RicF C138A or S^*f*^	8.7, 7.4, 7.7 (**7.9**)
RicF C144A C146A	4.6, 4.9 (**4.8**)

a In each row, the value in parentheses and boldface represents the average of the preceding values.

b All the proteins were purified and reconstituted anaerobically unless otherwise noted.

c Proteins purified aerobically and reconstituted anaerobically.

d Proteins purified from a *suf-*overexpressing E. coli strain anaerobically without reconstitution.

e The presence of 8 atoms per (RicAFT)*^Bsu^* complex was previously documented (5).

f The corresponding values in the second column represent results of cysteine-to-alanine mutations except for the values in italics, which represent results of a cysteine-to-serine mutation.

10.1128/mBio.01841-19.6 FIG S6 Secondary structure prediction using PSIPRED (http://bioinf.cs.ucl.ac.uk/psipred/) for RicF, RicA, and RicT, all from B. subtilis. Relevant C residues are underlined. Download FIG S6, PDF file, 2.0 MB. Copyright © 2019 Adusei-Danso et al. 2019 Adusei-Danso et al. This content is distributed under the terms of the Creative Commons Attribution 4.0 International license.

As noted above, the RicAF heterodimer cannot accept an Fe-S cluster. The data presented so far are most simply explained by a model (see Fig. 11) in which cluster 1 is coordinated entirely by C161, C166, C198, and C203 from RicT, not only when RicT is expressed by itself but also in the context of the ternary complex. This predicts that a ternary complex in which an essential cluster 1 ligand on RicT was changed to serine would accept 4 atoms of Fe per complex rather than 8 as in the wild-type complex. To test this, we purified and reconstituted a (RicAFT)*^Ban^* complex containing RicT C161S. In agreement with this prediction, the purified mutant complex contained an average of 4.5 Fe atoms (Table 2).

Because RicAF cannot accept an Fe-S cluster, while RicT carries a single cluster, it is likely that cluster 2 is coordinated by residues contributed by both RicT and RicAF. To test the possible contribution of RicT C167 and C204 to ligation of cluster 2, mutations at these sites were introduced into (RicAFT)*^Ban^*. After reconstitution, the C167S complex accepted an average of 4.2 Fe atoms per molecule of protein and the C204S complex accepted 8.0 (Table 2), suggesting that C167 but not C204 was involved in coordinating cluster 2 (see Fig. 11).

Four cysteine residues located near the C terminus of RicF are conserved in B. subtilis, G. stearothermophilus, and B. anthracis and are plausible candidates for ligation to cluster 2 (Fig. S5C). To assess the contributions of the conserved RicF cysteine residues, mutant (RicAFT)*^Ban^* complexes in which C134, C138, C144, or C146 was converted to serine or alanine were purified and subjected to reconstitution. A double mutant (C144A C146A) complex as well as the RicAFT complexes with individual C134A, C134S, C146A, and C146S replacements carried only about 4 Fe atoms, while RicF C138A, C138S, C144A, and C144S mutations had minor effects (Table 2). Taken together, these data support a model (see Fig. 11) in which cluster 2 is coordinated by RicT C167, RicF C134, and RicF C146, leaving the identity of the presumed fourth cluster 2 ligand unidentified thus far. RicA*^Bsu^* contains a single cysteine residue (C141). However, RicA*^Ban^* carries two cysteine residues very close to the C terminus of RicA (Fig. S5D) and we therefore changed both of these simultaneously to serine in the (RicAFT)*^Ban^* complex. Table 2 shows that the mutant complex carries an average of 4.5 Fe atoms, strongly supporting the conclusion that one of these two cysteine residues coordinates cluster 2 and that C141 is the ligating residue in the corresponding B. subtilis complex.

**FIG 7:**
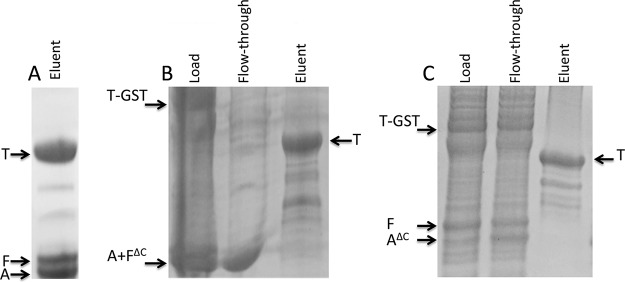
C-terminal truncations of RicA and RicF (A^ΔC^ and F^ΔC^) prevent the stable assembly of the RicAF heterodimer with RicT. (A) Results of SDS-PAGE analyses of elution of wild-type G. stearothermophilus complex from a glutathione affinity column after cleavage with PreScission protease. RicT was eluted together with RicA and RicF. (B and C) In contrast, when the 25 C-terminal residues were deleted from RicF (B) or the 23 C-terminal residues were deleted from RicA (C), RicA and RicF were recovered in the flowthrough fraction and only RicT was eluted after protease cleavage. RicA and RicF^ΔC^ proteins do not resolve in SDS-PAGE.

Because cluster 2 is coordinated by residues located in RicT and close to the C termini of RicF and RicA, residues in both C-terminal tails might be expected to make important contacts with RicT. In fact, when the 25 residues from the tail of RicF were deleted (RicF^ΔC^), a (RicA RicF^ΔC^T)*^Gst^* complex could not be isolated. Expressed in E. coli with a GST tag on RicT, all three wild-type proteins eluted together from the glutathione column when the tag was cleaved off with HRV-3C protease, as expected (Fig. 7A). In contrast, RicA and RicF^ΔC^ did not elute with RicT but were found in the flowthrough fraction (Fig. 7B). A similar result was obtained with a (RicA RicF^ΔC^T)*^Bsu^* expression construct (A. Tanner, V. Carabetta, and D. Dubnau, not shown), and it seems likely that the C-terminal tail of RicF makes important contacts with RicT. Examination of an equivalent construct in which 23 residues from the C-terminal tail of RicA*^Gst^* were deleted similarly demonstrated a loss of association between RicA^ΔC^F and RicT (Fig. 7C). As shown in Fig. 3, the C termini of RicA and of RicF emerge from the RicAF structure on opposite faces of the dimer, and Fig. 7 suggests that RicT may require contact with both tails for stable association with the heterodimer (see Fig. 11).

### Phenotypic effects of RicT C-to-S mutations.

To gain insights into possible roles for the Fe-S clusters, we examined phenotypic consequences of altering the ligating cysteine residues of RicT, RicA, and RicF. For this, we built individual cysteine-to-serine mutations into *ricT*, *ricA* and *ricF* at their native loci in B. subtilis, ensuring that the mutant genes were regulated normally at least on the level of transcription. Before presenting the results, it is important to present confounding considerations that affect the interpretation of phenotypes obtained with such mutations. First, all of the cysteine residues that coordinate the clusters are highly conserved. Although it is likely that a given phenotypic change is due to the absence of a cluster, it is also possible that the mutation has some other effect, perhaps on protein conformation, although the mutant proteins are soluble and complex formation is undisturbed. Also, since the developmental pathways of competence, biofilm, and spore formation involve the actions of Ric proteins at multiple steps (7), it is possible that individual mutations would differ in their ultimate phenotypic effects, particularly if both Fe-S clusters are not needed for all the functions of the proteins.

**FIG 8:**
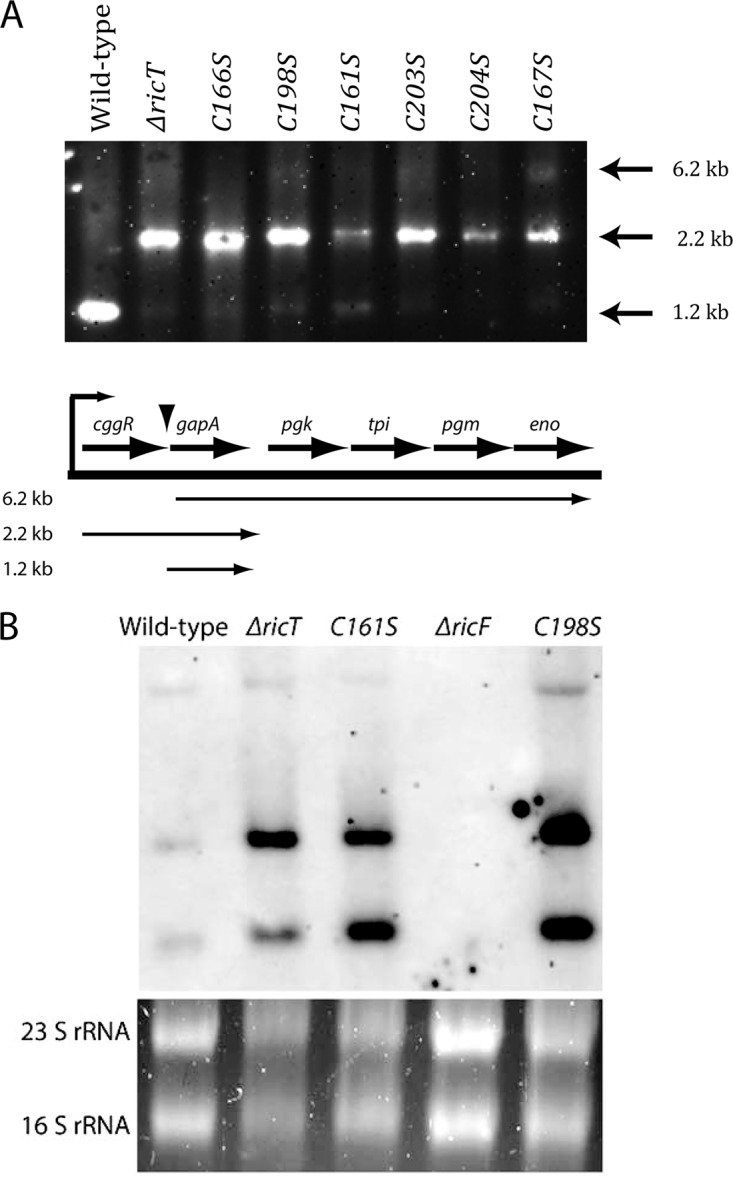
Effects of RicT cysteine-to-serine mutations on maturation of the *cggR-gapA* transcript (A) and on the stability of the *ricF* transcript (B). The probes for these Northern blots were complementary to *gapA* (A) and *ricF* (B) sequences. (A) Map of the expected transcripts (15). (B) Ethidium bromide-stained agarose gels containing the samples used for Northern blotting as loading controls, with the positions of major rRNA species indicated. In panel B, two distinct transcripts complementary to the ricF probe were detected.

**FIG 9:**
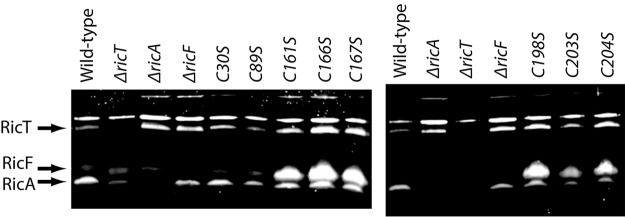
Western blot using extracts of the indicated mutants. The antibody was raised against the ternary complex, and the positions of the three Ric proteins are marked. Two separate gels are shown. All of the cysteine substitutions were located in RicT.

We first examined mRNA maturation in the mutant strains. It has been shown that deletion of each of the *ric* genes causes a defect in the maturation of the *cggR-gapA* transcripts (6, 8). These transcripts are normally cleaved between *cggR* and *gapA*, resulting in stabilization of the *gapA* mRNA (15). Our Northern blotting results confirm that the deletion of *ricT* caused a profound reduction in processing, as reported previously (6, 8), and show further that each of the point mutations in *ricT* had the same effect (Fig. 8A), suggesting that the clusters are essential for RNA maturation. This effect was not due to instability of the point mutant RicT proteins, as shown by Western blotting (Fig. 9). Although the mutant RicT signal was not significantly reduced by the cysteine-to-serine mutations, the amount of RicF protein was markedly increased in each point mutant. A much less dramatic increase was sometimes observed in the *ricT* deletant, although this is not apparent in Fig. 9. This overabundance of RicF was not due to increased transcription, as shown using *luc* fusions to the *ricF* promoter, which were expressed similarly in all the mutant strains (Fig. 4A); rather, it was caused by accumulation of the *ricF* transcript in C161S and C198S (Fig. 8B). Because the *ricF* transcript was also overabundant in the *ΔricT* strain (Fig. 8B), it is likely that RicT is required to modulate the turnover of the *ricF* transcript and that the point mutant RicT proteins are defective in this role. These results are most simply explained by a role for at least cluster 1 for both *cggR-gapA* RNA maturation and turnover of the *ricF* transcript, the latter causing an excess accumulation of RicF protein in the mutant strains. Although, as noted above, only a small increase of RicF was occasionally detected by Western blotting in a *ΔricT* strain, the *ricF* transcript was also stabilized in this strain (Fig. 8B). It is likely that in the absence of its binding partner (RicT), the RicF protein is partially destabilized, mitigating the effect of increased transcript stability on protein abundance.

**FIG 10:**
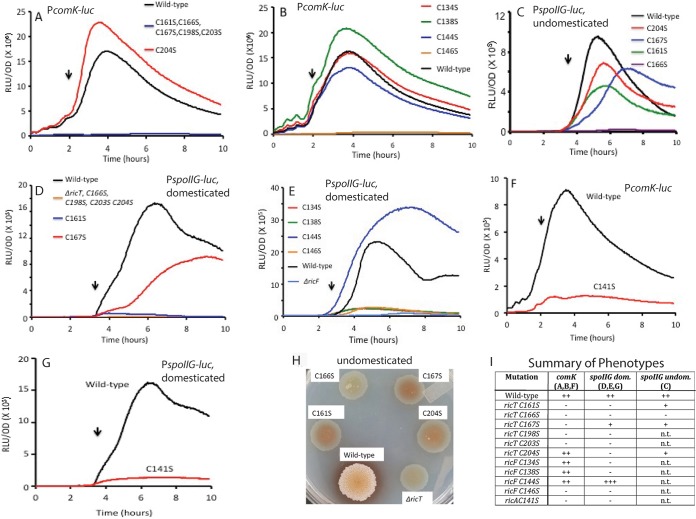
Effects of cysteine-to-serine mutations on P*comK-luc* and P*spoIIG-luc* expression and on biofilm formation after growth on an MsGG agar plate. All of the panels other than panels C and H show results determined in the domesticated IS*75* background. (A) Effects of RicT mutations on P*comK-luc* expression. (B) Effects of RicF mutations on P*comK-luc* expression. (C) Effects of RicT mutations on P*spoIIG* expression in the undomesticated ATCC 3610 background. (D) Effects of RicT mutations on P*spoIIG* expression in IS*75*. (E) Effects of RicF mutations on P*spoIIG* expression. (F) Effect of the RicA C141S mutation on the expression of *comK*. (G) Effect of the RicA C141S mutation on the expression of *spoIIG* in the domesticated background. (H) Biofilm formation with RicT mutations in ATCC 3610. (I) Summary of mutational effects displayed in the panels indicated in parentheses in the column headings. n.t.: not tested. The vertical arrows mark *T*_0_, the time of transition to the stationary phase.

We next turned to additional phenotypic effects of the cysteine substitution mutations. The results shown in Fig. 10A reveal that mutations in the four residues that coordinate cluster 1 (C161S, C166S, C198S, and C203S of RicT) obliterate expression of P*comK*, as does C167S, which coordinates cluster 2. Alanine substitutions of these RicT residues also eliminated expression from P*comK* (not shown). In contrast, serine substitution at C204, a RicT site that does not coordinate either cluster, was found to consistently elevate the expression of P*comK-luc*. Panel B of Fig. 10 shows that of the two RicF residues that coordinate cluster 2, the mutation of C146 decreased the expression of *comK* whereas mutation of C134 had no effect.

Panel C of Fig. 10 shows that in ATCC 3610, the RicT C161S, C167S, and C204S mutations affected the expression of *spoIIG* to approximately the same extent as the simultaneous deletion of all three *ric* genes (compare Fig. 5B). However, the *ricT* C166S mutation had an extreme effect on *spoIIG* expression compared to the other RicT cysteine residues (Fig. 10C). It is possible that C166 is needed for stimulation of the phosphorelay apart from its role in coordinating cluster 1. This is consistent with the failure of the C166S mutant, like the *ΔricT* strain, to produce pullcherrimin as shown by the absence of red pigment in Fig. 10H, given the observation that transcription of the genes for pulcherrimin synthesis is controlled by the Spo0A-P-regulated repressor AbrB (16). The presence of defective SigH in the domesticated strain background provided a more sensitive readout of the mutational effects on expression from P*spoIIG.* Accordingly, in the domesticated laboratory strain, these substitutions exhibited more-extreme effects (Fig. 10D), with the exception of C167S, which had a lesser effect than the other mutations. Also, the RicF C134S, C138S, and C146S mutations had major effects in the domesticated strain, lowering *spoIIG* expression profoundly, whereas the mutation at C144, which does not ligate a cluster, did not (Fig. 10E). The C161S, C166S, C167S, and C204S strains all formed flat featureless patches on MsGG agar (5 mM potassium phosphate [pH 7], 100 mM MOPS [morpholinepropanesulfonic acid; pH 7], 2 mM MgCl_2_, 700 μM CaCl_2_, 50 μM MnCl_2_, 50 μM FeCl_3_, 1 μM ZnCl_2_, 2 μM thiamine, 0.5% glycerol, 0.5% glutamate, 50 μg/ml tryptophan, 50 μg/ml phenylalanine), resembling the appearance of the *ΔricT* strain (Fig. 10H), consistent with a role for at least cluster 1 in biofilm formation. These same mutations profoundly decreased expression from the *tapA* promoter (Fig. 6B). Finally, the substitution of serine at RicA C141, which ligates cluster 2, profoundly affected both *comK* expression and *spoIIG* expression (Fig. 10F and G). Although the RicT C204S and RicF C138S mutations did not affect ligation of the Fe-S clusters, they showed clear phenotypic effects, presumably due to conformational or other effects that compromised function.

Although there are unexplained but not unexpected complexities in the patterns of phenotypic effects, these results, taken together, are most simply explained as showing that the RicAFT Fe-S clusters are needed for the maturation of *cggR-gapA* RNA; for the turnover of *ricF* transcripts; and for the proper regulation of biofilm formation, competence, and sporulation.

## DISCUSSION

**FIG 11:**
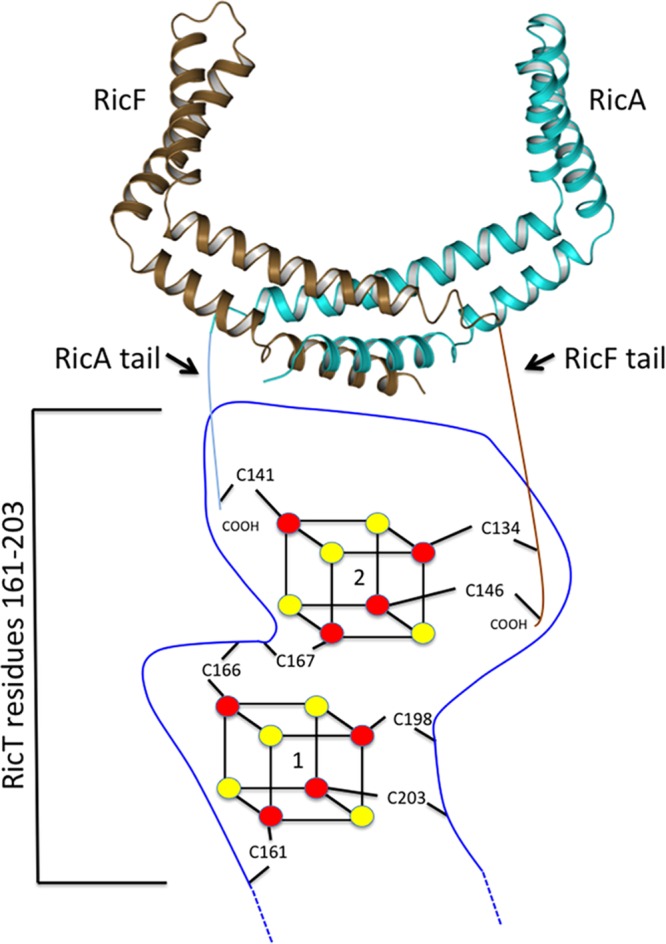
A schematic representation of the organization of the Fe-S clusters on the ternary Ric complex. RicA and RicF are depicted in cyan and brown, respectively, and the ligating residues for clusters 1 and 2 are shown. The blue line represents the RicT sequence that is proposed to be “stapled” by cluster 1, presenting this portion of RicT for interactions with the C-terminal portions of RicA and RicF.

The present report advances our understanding of the Ric proteins, which are widespread among the firmicutes and are associated with major and pleiotropic phenotypes in B. subtilis. Importantly, we have shown that the complex containing RicA, RicF, and RicT is a 1:1:1 heterotrimer, formed by association of a RicT monomer with a RicAF heterodimer. The resulting trimer carries two [4Fe-4S]^+2^ clusters (5), one of which is ligated entirely by RicT residues and can be maintained on RicT in the absence of RicAF. This cluster is coordinated by four of the six conserved cysteine residues of RicT, namely, C161, C166, C198, and C203. The second cluster is coordinated by C167 from RicT, by C134 and C146 from RicF, and by C141 from RicA, placing the two clusters at an interface of the three proteins and in close proximity to one another because C166 (cluster 1) and C167 (cluster 2) are adjacent residues. A schematic illustrating the proposed arrangements of the clusters in presented in Fig. 11, suggesting the presence of what may be an active center for the ternary Ric complex at the interface of the three subunits.

We have generated structures of a RicA homodimer and of a likely tetramer formed from RicA and RicF, and we have shown that several association states of the three proteins can exist *in vitro*. These are also likely to exist *in vivo*, based on the unequal molar amounts of the three proteins (Table 1) and their distinct expression patterns (Fig. 4). RicA can exist in complex with itself to form a homodimer or with RicF to form a tetramer. Using Pisa analysis (9), we have identified buried residues at the RicA-RicA and RicA-RicF interfaces, which we conservatively define as those with >20% buried surface area. Of 36 buried RicA residues in RicA-RicA, 28 are also buried in RicA-RicF. Only 29 RicF residues are buried at the RicA-RicF interface, and 9 (31%) of these residues are conserved in the RicA sequence compared to an overall level of identity of the two proteins of about 20%. These results suggest that the two proteins may have diverged from a common ancestor with strong selection pressure to maintain both the RicA/RicF and RicA/RicA interfaces. Because there is no evidence for a RicA tetramer, it would appear that the association of RicA with itself precludes tetramerization and, indeed, the tetramerization interface between RicAF dimers involves only RicA-RicF contacts (rather than RicA-RicA or RicF-RicF contacts) (Fig. 3B). Another type of exclusion is evident when RicT is present. RicA does not associate with itself but rather with RicF to form RicAFT, as if RicT cannot associate with the RicA homodimer or the RicAF tetramer. An explanation of this exclusive arrangement awaits elucidation of the structure of RicAFT. It may be that the various concentrations in the cell of the three proteins during growth and in different media (Table 1) dictate the probabilities of formation of these alternative complexes, resulting in distinct independent regulatory consequences.

RicA and RicF have C-terminal tails that are predicted to be unstructured or to form coils in the individual proteins (see Fig. S6 in the supplemental material), and the residues from 125 to the C terminus in the deposited structure of RicA (PDB ID 2PIH) are indeed disordered. With the tails deleted, RicAF^ΔC^ and RicA^ΔC^F failed to establish stable complexes with RicT, suggesting that RicT contacts residues in the C-terminal tails of both RicA and RicF, introducing order in them. Our inability to demonstrate stable individual complexes of RicT with only full-length RicF or only full-length RicA (Fig. 1) is consistent with the idea that RicT associates stably with RicA or RicF only when both proteins are present in a heterodimer, making specific contacts with each of them. Also consistent are the conclusions that RicF C134 and C146 tail residues coordinate cluster 2 together with RicT C167, placing this cluster at the RicT-RicF interface, and that RicA C141 also coordinates cluster 2, placing RicT-RicA contacts close to the RicT-RicF interface. Although this intimate arrangement of two Fe-S clusters at the interface of three proteins is, to our knowledge, unique, other examples have been reported of Fe-S clusters ligated to residues in different subunits of a binary complex (17–21).

It is notable that there are two pairs of adjacent conserved cysteine residues in RicT. We are aware of no documented cases in which adjacent cysteine residues coordinate the same Fe-S cluster, with the possible exception of the N2 cluster in complex I (NADH:ubiquinone oxidoreductase) (22). Mutation of either of the vicinal cysteines in that case caused loss of the cluster, whereas only one mutation in each pair of cysteines (C166 and C203) in the Ric complex caused loss of cluster 1. As already noted, the second cysteine in the first pair (C167) ligates cluster 2. Whereas the clusters are not essential for the *in vivo* stability of the Ric proteins either in B. subtilis or under conditions of expression in E. coli and whereas their absence does not noticeably affect the stability of the ternary complex *in vitro*, the reverse dependence is observed: association of the three protomers is needed for cluster 2 formation.

In view of the Ric protein requirements for RNA processing (6, 8), it is interesting that a number of other proteins mediating nucleic acid transactions are known to carry Fe-S clusters. For example, several radical SAM (S-adenosyl methionine) enzymes covalently modify tRNA and rRNA (23, 24), and the gene for one of them, *miaB*, is adjacent to *ricA*, with fairly well conserved synteny (7). Other nucleic acid transaction enzymes carry Fe-S clusters (25, 26), including helicases, eukaryotic primases, DNA glycosylases, and the ATP-dependent helicase/nuclease AddAB, which is required for double-strand DNA break repair in some Gram-positive bacteria. AddAB carries a single [4Fe-4S] cluster that serves as a “molecular staple,” coordinating cysteine residues that are separated in the primary sequence and thereby facilitating the correct folding and presentation of the intervening sequence (27). In RicT, the C161/166 and C198/203 pairs that coordinate cluster 1 are separated by 30 residues that are predicted to be in a coiled or disordered conformation (Fig. S6). An attractive possibility is that cluster 1 secures the base of a loop to present the intervening sequence for interactions with RicAF (Fig. 11).

Despite our increased descriptive understanding of the *ric* genes and proteins, the biofilm and K-state deficiencies of *ric* mutants are not fully understood. It has been suggested that the *sinR* transcript, which encodes a repressor of important biofilm genes, is stabilized in the absence of RicA or RicF (6, 8). It has alternatively been suggested that the phosphorelay, which is needed to counter the activity of SinR, is less active in the absence of Ric proteins (4, 5). Neither of these competing hypotheses is fully explanatory, because even in a *ΔsinR* strain, the transcription of a biofilm gene (Fig. 6A) and the maturation of biofilms (7) are severely impacted when *ric* genes are inactivated.

Ric protein requirements for a given phenotype do not necessarily imply direct involvement, although regulation of the phosphorelay represents one case where *in vitro* biochemical studies do suggest a direct role (5). Although the Ric proteins are required for RNA maturation, the evidence that they are directly involved derives from their association with the RNase Rny, based on positive signals obtained with RicF and RicA in bacterial 2-hybrid experiments (6) and pulldown results in B. subtilis with RicF but not with RicA in one case (6) and with RicT and not with RicA or RicF in another (4). Also, the membrane association of RicT is dependent on the presence of Rny (8). Although the evidence favoring a direct involvement in RNA maturation is highly suggestive, the possibility that other mechanistic roles exist for the pleiotropic Ric proteins, whether they are together in a ternary complex or in other associative forms, has not been excluded.

A related issue concerns the role(s) of the Fe-S clusters, aside from their possible contribution to structure. Since the phenotypes of *Δric* strains are similar to those of strains with mutations in cluster-ligating residues, it is likely that the clusters are needed for biofilm formation, competence expression, and sporulation as well as for maturation of the *cggR-gapA* transcript and for turnover of the *ricF* mRNA. It is likely that understanding the roles of the Fe-S clusters will eventually provide insight into Ric protein activity and associated mechanisms; an intriguing possibility is that the clusters transduce metabolic signals related to the regulation of RNA maturation and to the phosphorelay. However, Fe-S clusters play many roles in nature, including roles as sensors of redox conditions and of iron availability, as mediators of Fe-S cluster assembly and repair, as mediators of electron transfer reactions, and as protein structural elements, and roles in catalysis, for example, in radical SAM enzymes and in aconitase. It is worth noting that all of the proteins with subunit-bridging Fe-S clusters that we have found in the literature seem to be themselves involved in cluster assembly or repair (17–21, 28), and this possibility must also be considered for the Ric proteins.

## MATERIALS AND METHODS

### Media, strains, and growth conditions.

All mutant strains of B. subtilis were constructed in IS*75* (*his leu met*) or ATCC 3610. B. subtilis was grown in Luria-Bertani medium (29), MsGG (1), competence medium (30), or Spizizen minimal medium (31) supplemented with glucose (0.5% [wt/vol]). After insertion, mutations were confirmed by sequencing. The firefly luciferase fusions to the *comK*, *tapA* and *spoIIG* promoters were constructed and assayed as described previously (32).

### Plasmid constructions and mutagenesis.

Cloning primers are listed in Table S2 in the supplemental material. For protein expression in E. coli, *ric* genes were cloned into the pQLINK plasmid (33) as described previously (5) and combined for coexpression using a previously published protocol (33). Mutagenesis was carried out using Change-It or Phusion site-directed mutagenesis kits (Thermo Fisher). Point mutations were moved into the chromosome of B. subtilis at the native loci using the pMiniMad2 plasmid as described previously (34).

10.1128/mBio.01841-19.8 TABLE S2 Cloning primers. Download Table S2, PDF file, 0.04 MB. Copyright © 2019 Adusei-Danso et al. 2019 Adusei-Danso et al. This content is distributed under the terms of the Creative Commons Attribution 4.0 International license.

### Analytical ultracentrifugation.

Samples were prepared in 50 mM sodium phosphate buffer (pH 7.5) containing 50 mM NaCl. All measurements were made at 40,000 rpm (RicAFT) and 35,000 rpm (RicT) at 20°C by UV intensity detection in a Beckman Optima AUC analytical ultracentrifuge at the Center for Analytical Ultracentrifugation of Macromolecular Assemblies at the University of Texas Health Science Center at San Antonio, using an An60Ti 4-hole rotor and standard 2-channel epon centerpieces with 1.2-cm path length (Beckman-Coulter). Two different concentrations were examined to determine if mass action effects were present in the concentration range of interest. The following samples and concentrations were measured: (i) RicAFT 1:1:1 (B. anthracis) at an optical density at 280 nm (OD_280_) of 0.696 (∼15 μM); (ii) RicAFT 1:1:1 (B. anthracis) at an OD_280_ of 0.393 (∼8.5 μM); (iii) RicT (G. stearothermophilus) at an OD_280_ of 1.03 (∼42 μM); (iv) RicT (G. stearothermophilus) at an OD_280_ of 0.38 (∼15 μM).

Sedimentation and diffusion transport in the ultracentrifugation cell are described by the Lamm equation, which can be solved using adaptive finite element methods (49, 50). Whole-boundary data obtained in SV experiments are fitted by linear combinations of such solutions using advanced optimization routines (35–37). All data were analyzed with UltraScan-III ver. 4.0, release 2577 (38, 39). The hydrodynamic corrections for buffer density and viscosity were estimated by UltraScan to be 1.0017 g/ml and 1.0046 cP. The partial specific volumes of the RicAFT complex (0.7382 ml/g) and RicT (0.7447 ml/g) were estimated by UltraScan based on their amino acid sequence by the use of methods analogous to methods outlined previously by Laue et al. (40).

SV data were analyzed according to a workflow described previously (41). Optimization was performed by 2-dimensional spectrum analysis (2DSA) (35) with simultaneous removal of temporally and radially invariant noise contributions (42). After inspection of the 2DSA solutions, a global 2DSA-Monte Carlo analysis was performed to calculate confidence limits for the determined parameters (36) and to obtain a model that described the two concentrations equally well (since there was no change in the sedimentation distribution upon dilution). The calculations are computationally intensive and are carried out on high-performance computing platforms (37). Integral sedimentation distributions were evaluated with the enhanced van Holde-Weischet method (43) to determine if shifts of sedimentation distributions occurred as a function of mass action.

### Acrylamide gel electrophoresis and Western blotting.

Resolution of the Ric proteins by SDS-PAGE presented reproducibility and resolution problems due to the high level of cysteine residue content and the closely similar masses of RicA and RicF. To mitigate these problems, the following procedure was adopted. Culture samples (1 ml) grown to the exponential or stationary phase in the specified media were harvested and washed in STM protoplasting buffer (25% sucrose, 50 mM Tris-HCl [pH 8], 5 mM MgCl_2_, and 50 mM NaCl plus protease inhibitors) and resuspended in STM buffer containing 0.3 mg/ml lysozyme, adjusted to equivalent cell densities based on Klett colorimeter values. After incubation at 37°C for 15 min, 5× cracking buffer (250 mM Tris-HCl [pH 8], 5% [wt/vol] SDS, 50% [wt/vol] glycerol, and 0.2% [wt/vol] bromophenol blue plus freshly added 5% β-mercaptoethanol [2-ME]) was added to the samples to obtain a 1× final concentration. The samples were held in a boiling water bath for 10 min and were then loaded on 15% acrylamide Bis-Tris gels (0.3 M Bis-Tris gel buffer [pH 6.5 to 6.8]). The gels were run using 2-(*N*-morpholino)ethanesulfonic acid (MES) running buffer plus sodium bisulfite (50 mM MES, 50 mM Tris, 1 mM EDTA, 0.1% SDS, and freshly added 5 mM sodium bisulfite) in both the cathode and anode chambers. After electrophoresis was performed, the gels were blotted to nitrocellulose membranes using a Transblot Turbo apparatus (Bio-Rad) and developed using rabbit antisera raised against the ternary Ric complex. The signals were visualized using ECL Prime Western blotting detection reagent (GE Healthcare).

To measure the cellular contents of the Ric proteins, lysates were subjected to Western blotting as described above, except that each gel contained a series of known quantities of purified Ric proteins. After blotting, digitized images of the membranes were quantified using Image J, standard curves were constructed, and the concentrations of the proteins in the lysate lanes were determined from these curves.

### Northern blotting.

B. subtilis strains were grown in 10 ml LB with aeration at 37°C until the late log phase of growth. Cells were harvested, and RNA was prepared using a FastRNA Pro Blue kit (MP Biomedicals) per the manufacturer’s instructions. The samples were processed in a FastPrep FP120 instrument for 3 cycles (45, 45, and 30 s) at a speed setting of 6.0. RNA concentrations were measured with a NanoDrop 1000 spectrophotometer (Thermo Scientific). RNA was resolved using agarose gel electrophoresis in 3-(N-morpholino)propanesulfonic acid (MOPS) buffer (0.2 M MOPS, 20 mM sodium acetate, 10 mM EDTA) with 1.5% agarose containing 5% formaldehyde. Electrophoresis was performed at 50 V for 50 min. The RNA loading buffer contained 50 μl 80% glycerol, 600 μl formamide, 200 μl formaldehyde, 240 μl 10× MOPS, 4 μl ethidium bromide, and a few crystals of bromophenol blue. All solutions were made with diethyl pyrocarbonate-treated water. For Northern analysis, ethidium bromide was omitted. RNA was transferred to Bright Star-Plus positively charged nylon membranes (Ambion) using passive (capillary) transfer. The membrane was subjected to UV cross-linking in a Spectrolinker XL-1000 UV cross-linker. The membrane was then prehybridized with DIG Easy Hyb (Sigma-Aldrich), hybridized with digoxigenin (DIG)-labeled RNA probe, washed, and incubated with anti-digoxigenin-AP fast atom bombardment (FAB) fragments (Sigma-Aldrich), and the bands were developed using disodium 3-{4-methoxyspiro [1,2-dioxetane-3,2′-(5′-chloro)tricyclo (3.3.1.13,7)decan]-4-yl}phenyl phosphate (CSPD; Sigma-Aldrich} per the manufacturer’s instructions. The bands were visualized using a Bio-Rad ChemiDoc MP imaging system. The digoxigenin-labeled RNA probes were transcribed from PCR products containing a T7 RNA polymerase promoter fused to *gapA* or *ricF* sequences by the use of T7 RNA polymerase and DigRNA labeling mix (Sigma-Aldrich) per the manufacturer’s instructions. The sequences of the *gapA* forward and reverse primers were 5′-CGATGCGCTAACCACGATGTTA and 5′-GAAATTAATACGACTCACTATAGGGACCATAACCATTGTAGAAAG, respectively. The *ricF* forward and reverse primers were 5′-ATGTATGCGACGATGGAATC and 5′-GAAATTAATACGACTCACTATAGGTCAGGACACTTTACATCCG, respectively. The T7 RNA polymerase promoter sequences are underlined.

### Luminometry for gene expression studies.

Cultures were grown in 96-well microtiter plates in an Envision 2104 (Perkin-Elmer) plate reader at 37°C with shaking. OD_600_ and luminescence determinations were performed at 2-min intervals throughout growth until the cultures were well into the stationary phase, as described previously (32).

### Protein purification and reconstitution and Fe determination.

Ric proteins were expressed in E. coli and purified either aerobically for crystallization or in an anaerobic Coy chamber for iron determination. Proteins were expressed individually or coexpressed from pQLink plasmids (33) in E. coli BL21(DE3)pLysE. Reconstitution with FeCl_3_ and Li_2_S was performed as described previously (5) except that a 9-fold molar excess of FeCl_3_ was used. Fe determination was carried out with the ferene assay (51) as previously described (5) using standard curves constructed using dilutions of iron standard for atomic absorption spectrometry (AAS) (Sigma-Aldrich) (10 g/liter). For calculation of Fe content, protein determination was done using the Bradford assay (52) with bovine serum albumen for construction of standard curves.

For expression and purification of RicA, RicAF, and RicAFT for Fe determination and for AUC, recombinant GST-RicT, GST-RicA, GST-RicAF, and GST-RicAFT plasmids were induced in E. coli BL21(DE3)pLysE. For crystallography, truncated versions of RicA (residues 1 to 122) and RicF (1 to 121) were used, removing residues that were predicted to be in a coiled conformation (Table S3). Protein expression and purification were performed as follows. Cultures were grown in LB media containing 100 μg/ml ampicillin to an OD_600_ of 0.6 to 0.8. Protein expression was induced with 500 μM IPTG (isopropyl-β-d-thiogalactopyranoside), and the cultures were grown overnight at 18°C. Harvested cells were resuspended in GST-lysis buffer A (50 mM Tris-HCl [pH 8.0], 200 mM NaCl, 5% glycerol, 0.1% Triton X-100, 5 mM dithiothreitol [DTT], and 1 mM phenylmethylsulfonyl fluoride [PMSF] with the addition of protease inhibitor tablets per the instructions of the manufacturer [Pierce]) and lysed using an Avestin Emulsiflex C5 cell disrupter (ATA Scientific Instruments). For anaerobic purification, a model 150 V/T ultrasonic homogenizer (BioLogics, Inc.) was used in a Coy chamber to disrupt the cells. The resulting lysate was clarified by centrifugation at 30,000 × *g* for 60 min in sealed tubes and subsequently incubated with Glutathione Superflow agarose resin (Thermo Scientific) for 2 to 4 h at 4°C. Anaerobic purification was carried out at room temperature. The resin was then washed extensively with low-salt buffer B (50 mM Tris-HCl [pH 8.0], 70 mM NaCl, 2 mM DTT) and then with high-salt buffer C (50 mM Tris [pH 8.0], 500 mM NaCl, 5 mM DTT) and again with buffer B. The GST tags were removed by overnight incubation of the washed resin with 3C protease (Thermo Fisher Scientific) at 4°C. That temperature was maintained in the anaerobic chamber by the use of a chilling block (Cole-Palmer, Inc.). Proteins were further purified by anion-exchange chromatography (Source 15Q; GE Healthcare Life Sciences) and eluted in a gradient with buffers B and D (50 mM Tris-HCl [pH 8.0], 1,000 mM NaCl, 2 mM DTT). Fractions eluting between 300 and 500 mM NaCl were concentrated to 2 to 4 mg/ml using 10-kDa molecular-weight-cutoff (MWCO) Amicon spin columns (Millipore) for RicT and RicAF and 30-kDa MWCO Amicon spin columns for the RicAFT complex. To prepare samples for AUC and crystallography, preparations were subjected to size exclusion chromatography in buffer E (20 mM Tris-HCl [pH 8.0], 150 mM NaCl, 1 mM DTT). A Superdex 200 10/30 column (GE Healthcare Life Sciences) was used for RicA, whereas we used a Superdex 200 16/70 column for RicT, RicAF, and RicAFT. The proteins were then concentrated again using Amicon spin columns (Millipore). Protein concentrations were measured on a NanoDrop 1000 spectrophotometer (Thermo Fisher Scientific) and calculated using a predicted extinction coefficient for absorbance at 280 nm (ExPASy ProtParam server) or using the Bradford assay (Bradford, 1976) with bovine serum albumin (BSA) as a standard.

10.1128/mBio.01841-19.9 TABLE S3 Mutagenic primers. Download Table S3, PDF file, 0.1 MB. Copyright © 2019 Adusei-Danso et al. 2019 Adusei-Danso et al. This content is distributed under the terms of the Creative Commons Attribution 4.0 International license.

For AUC experiments, RicT was expressed from a recombinant His_6_-Sumo-RicT plasmid in E. coli BL21(DE3) pLysE (Thermo Fisher Scientific). Cultures were grown in 2× TY medium (16 g tryptone, 10 g yeast extract, 5 g NaCl) or lysogeny broth (LB) containing 100 μg/ml ampicillin to an OD_600_ of 0.6 to 0.8. Protein expression was induced with 500 μM IPTG, and cultures were grown overnight at 18°C. Harvested cells were resuspended in lysis buffer F (50 mM Tris-HCl [pH 8.0], 200 mM NaCl, 5% glycerol, 0.1% Triton X-100, 5 mM imidazole [pH 8.0], 5 mM 2-ME, 1 mM PMSF) and lysed by two passages through a cell disrupter. The resulting bacterial lysate was clarified by centrifugation at 30,000 × *g* for 60 min and subsequently passed over His60 Superflow Ni resin (TaKaRa Bio USA) equilibrated in buffer G (50 mM Tris-HCl [pH 8.0], 200 mM NaCl, 10 mM imidazole [pH 8.0], 5 mM 2-ME). The resin was washed with buffer G and was eluted with an increasing-step gradient of imidazole in buffer G. Fractional purity was evaluated using SDS-PAGE. The highest-purity fractions were pooled and dialyzed overnight against buffer H (70 mM NaCl, 50 mM Tris [pH 8.0], 5 mM 2-ME). His_6_-Ulp1 SUMO protease was added to the pooled fractions prior to dialysis to cleave the His_6_-SUMO affinity tag. Following cleavage, the dialyzed sample was reapplied to a fresh His60 Superflow resin to remove the majority of the His_6_-SUMO tag and His_6_-Ulp1, with the flowthrough containing RicT. The flowthrough fraction was then passed over a SourceQ anion exchange column (GE Healthcare). RicT eluted in the SourceQ column was free of all significant contaminants. Following anion exchange, the pure fractions of RicT were pooled and concentrated using a 10-kDa MWCO Amicon spin column (Millipore). For analysis, the protein was loaded onto a Superdex 200 16/70 column (GE Healthcare) equilibrated with buffer I (20 mM Tris [pH 8.0], 100 mM NaCl, 5 mM DTT). Following size exclusion chromatography, RicT was concentrated to 10 mg/ml and used for subsequent crystallization trials and biochemical studies.

For overexpression of RicT in a strain containing pSuf (a kind gift from Sameh H. Abdelwahed), pQLink RicT and the pSuf plasmid were cotransformed into E. coli BL21pLYS and a single fresh colony was used to inoculate 200 ml of 2× TY medium supplemented with 30 μg/ml chloramphenicol and 100 μg/ml ampicillin. After growth overnight at 37°C, 2 ml of this starter culture was used to inoculate 4 liters (2 liters in each of two 5-liter flasks) of the same medium and the cultures were grown at 37°C with shaking to an OD_600_ of 0.6 to 0.8. The cultures were chilled, and each flask was supplemented with a 200 mM concentration of ferrous ammonium sulfate and a 200 mM concentration of l-cysteine and with 1 mM IPTG. The cultures were then grown overnight at 16°C on a rotary shaker (220 rpm). The cells were then harvested by centrifugation, and RicT was purified from the dark brown pellets as described above.

### Selenomethionine (Se-Met) labeling of the RicAF complex.

Protein labeling was carried out using media supplied by Molecular Dimensions (United Kingdom). The concentration of Se-Met was maintained at about 25 mg/liter. Initially, the primary culture was grown in LB medium overnight. The next morning, cells were harvested by centrifugation at 4,500 rpm for 20 min. The pellet obtained was resuspended in the complete Se-Met medium, and the process was repeated once again to remove any traces of LB medium. After inoculation of the secondary Se-Met complete medium when the OD_600_ reached 0.7 at 37°C, cells were induced with 0.5 mM IPTG and shaken for another 16 h at 18°C. Cells were harvested at 4,500 rpm for 20 min and stored at −80°C for downstream processing. Further purification was performed as described above for the native RicAF and RicA proteins.

### RicA and RicAF crystallization, X-ray diffraction data collection, and crystal structure determination.

RicA and RicAF crystallization conditions were identified in a high-throughput screen using purified RicAF (containing RicA and RicF residues 1 to 122 and 1 to 121, respectively). Initially, the composition of the RicA and RicAF crystals was determined by SDS-PAGE analysis of crystals washed in their crystallization reservoir solutions. RicA and RicF placement in the RicAF complex was validated by the positions of the selenium atoms in a model-phased anomalous-difference map of selenomethionyl-derivatized RicAF. Optimized RicA crystals were grown using the hanging drop vapor diffusion method and by mixing equal volumes of protein (10 mg/ml) and mother liquor containing 100 mM MES (pH 6.0), 150 mM NaCl, and 65% (vol/vol) MPD (Hampton Research) at 20°C. RicAF crystals were grown using the hanging drop vapor diffusion method and by mixing a protein solution (8 mg/ml) with mother liquor containing 18% (vol/vol) polyethylene glycol (PEG) 500 MME (Molecular Dimensions), 8% (vol/vol) MPD, 100 mM sodium nitrate, 100 mM MOPS, and sodium HEPES (pH 7.3). Prior to collection of X-ray diffraction data, RicA crystals were directly harvested and frozen in liquid nitrogen, while an additional amount of 17% (vol/vol) PEG 500 MME was added to the original crystallization mixture before flash-freezing of RicAF crystals in liquid nitrogen was performed. All diffraction data were collected on nitrogen-cooled crystals at 100 K at the Stanford Synchrotron Radiation Lightsource (SSRL) using beamlines 9-2 and 14-1 and processed using the HKL software package (44).

The initial phases for the RicA structure were determined in Phaser (45) using the molecular replacement method and the unpublished structure of YmcA (PDB ID 2PIH) as a search model. The RicA model (amino acids 1 to 122) was generated using iterative rounds of model building in Coot (46) and refinement in Refmac5 (47). One chlorine atom was built into clear electron density during the final stages of refinement. Similarly, the initial phases for the RicAF structure were obtained in Phaser (45) using the molecular replacement method and the structure of RicA converted to polyalanine as a search model. The final RicAF model contained residues 3 to 119 and residues 6 to 120 and was generated using iterative rounds of model building in Coot (46) and refinement in Buster (48). Data collection and refinement statistics are shown in Table S4.

10.1128/mBio.01841-19.10 TABLE S4 Data collection and refinement statistics. Download Table S4, PDF file, 0.1 MB. Copyright © 2019 Adusei-Danso et al. 2019 Adusei-Danso et al. This content is distributed under the terms of the Creative Commons Attribution 4.0 International license.

### Visualization of biofilms.

Bacteria were grown overnight in LB, and 5-μl aliquots were spotted on MsGG agar plates that had been allowed to dry overnight at room temperature. The plates were then incubated at 30°C and photographed after 3 days.

### Data accessibility.

Atomic coordinates and structure factors for RicA and RicAF have been deposited in the Protein Data Bank under accession numbers 6PRH and 6PRK.
