# Supplementary figures and images for: Structure-Function Studies of the Bacillus subtilis Ric Proteins Identify the Fe-S Cluster-Ligating Residues and Their Roles in Development and RNA Processing
Source: mBio. 2019 Sep 17;10(5):e01841-19. doi: 10.1128/mBio.01841-19 (PMC6751060; doi:10.1128/mBio.01841-19)

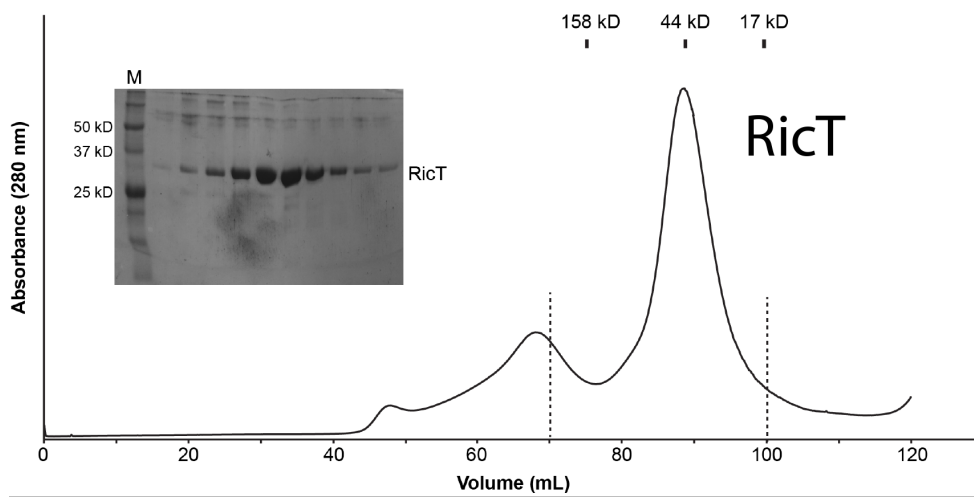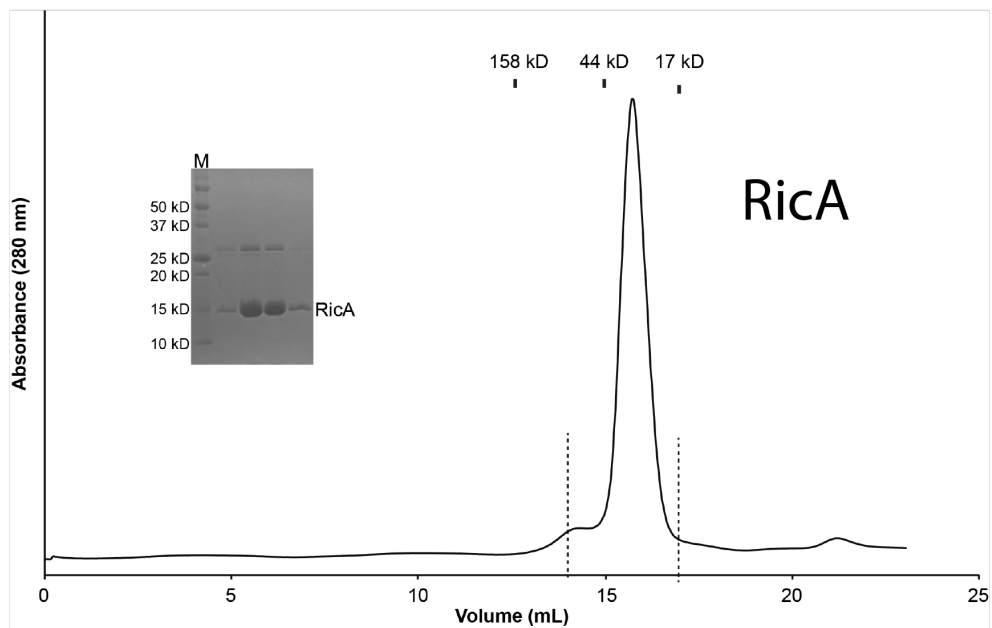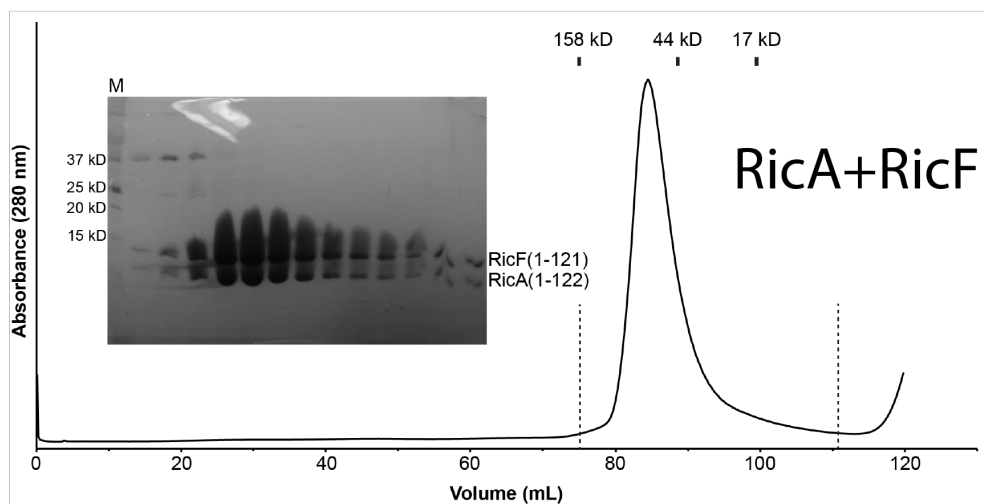

Supplement: FIG S1 [file mBio.01841-19-sf001.pdf]

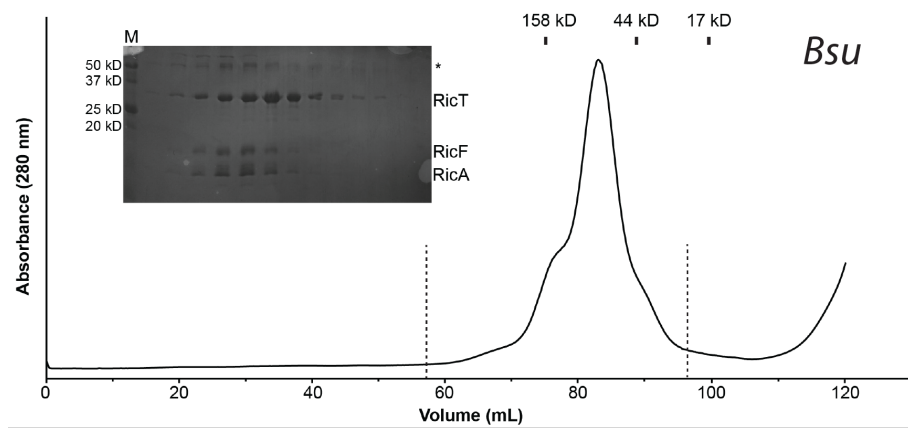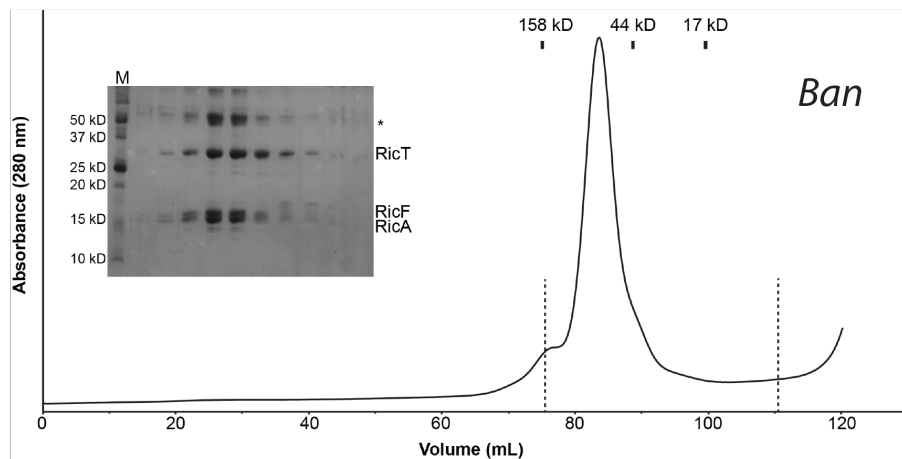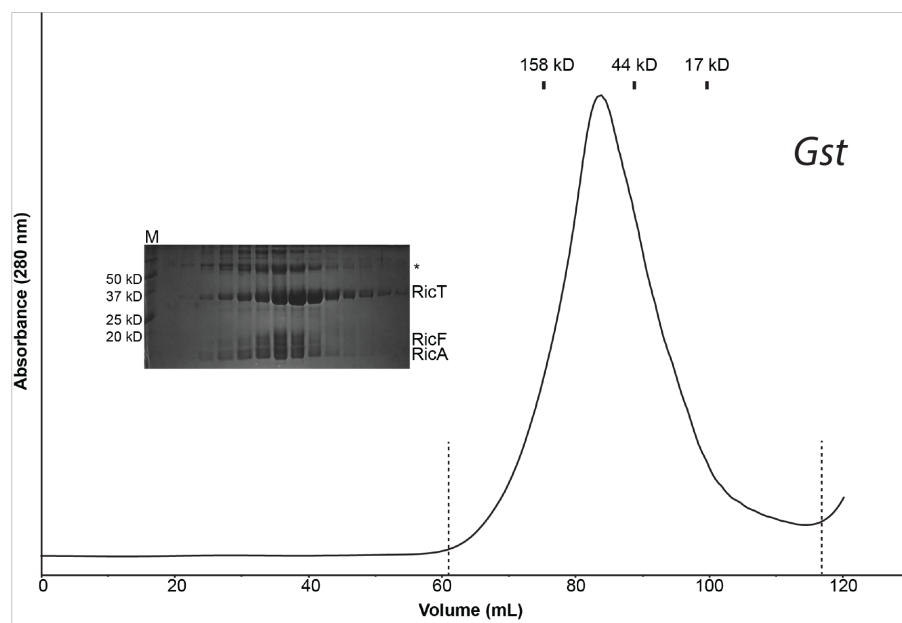

Supplement: FIG S2 [file mBio.01841-19-sf002.pdf]

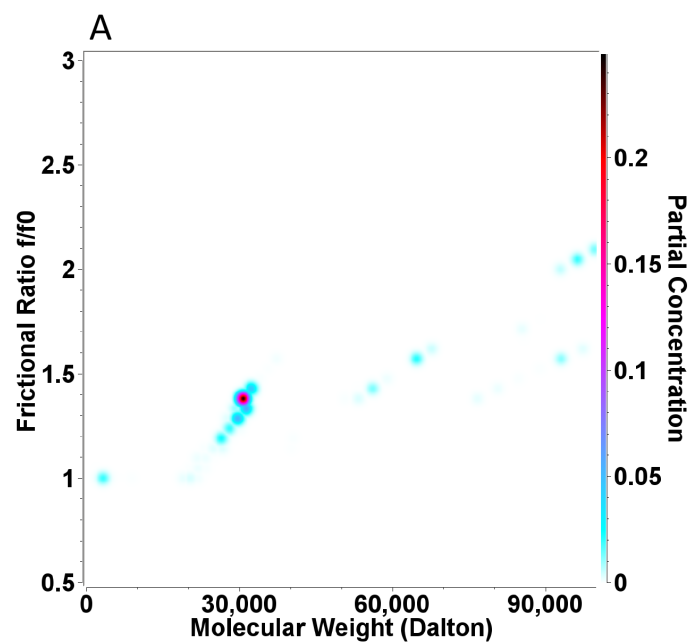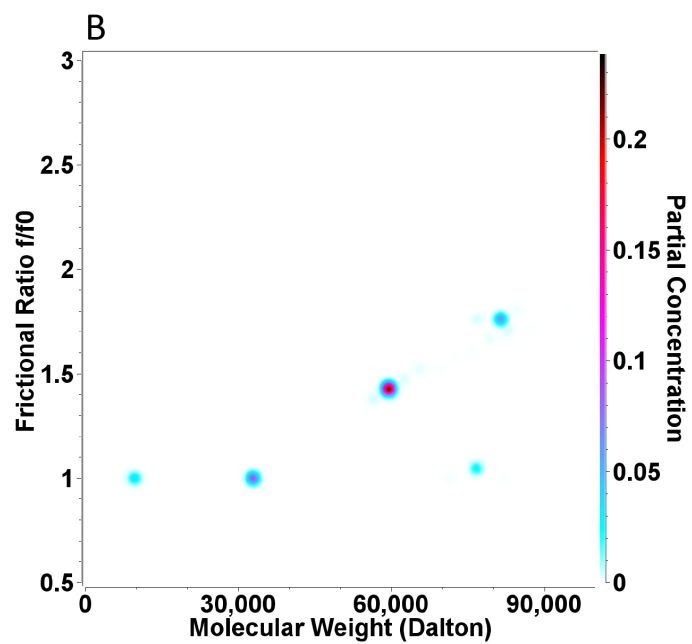

Supplement: FIG S3 [file mBio.01841-19-sf003.pdf]

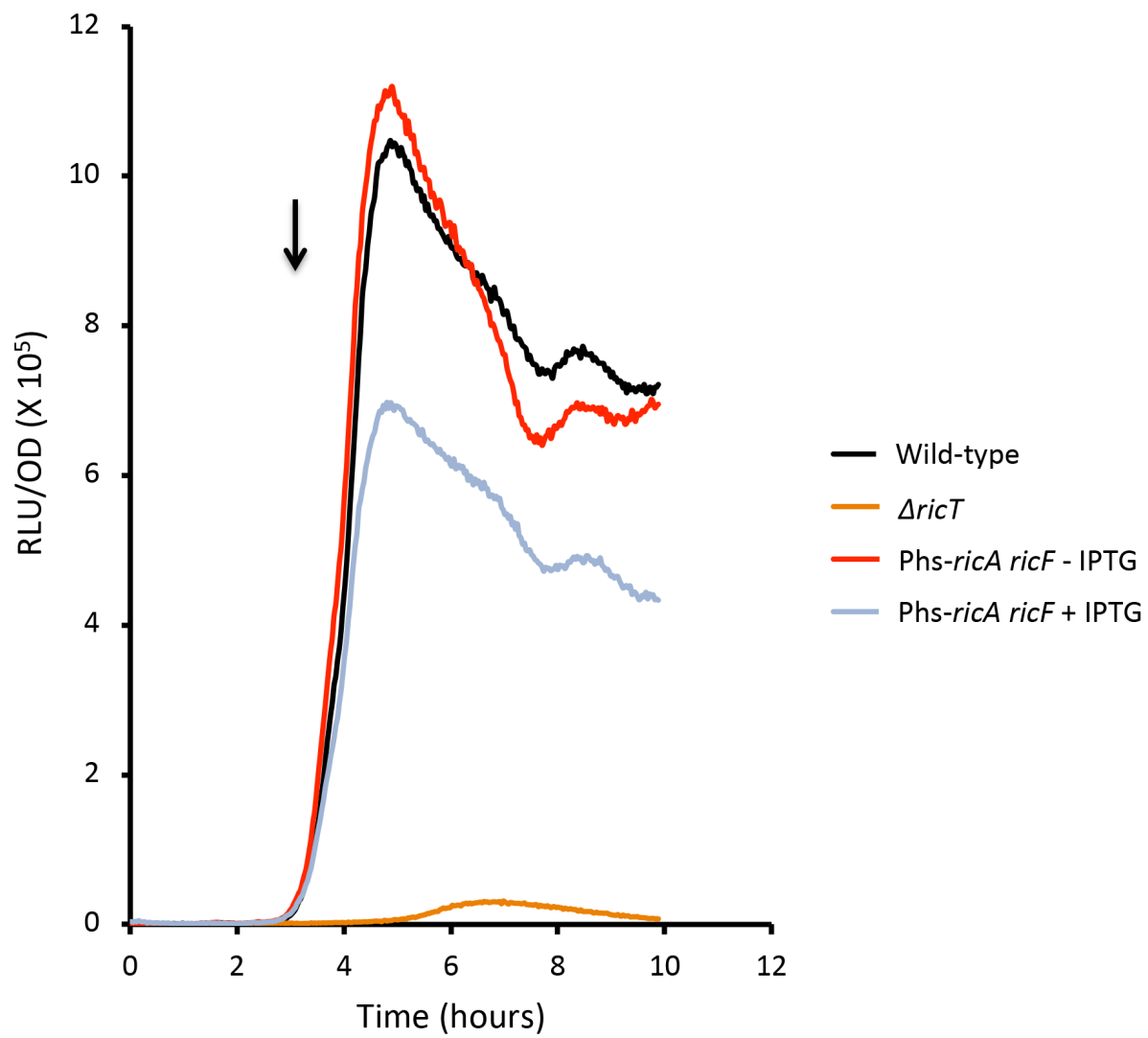

Supplement: FIG S4 [file mBio.01841-19-sf004.pdf]

## RicA

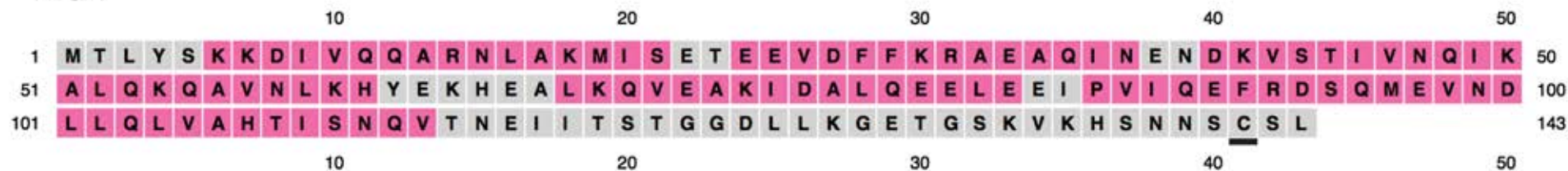

## RicF

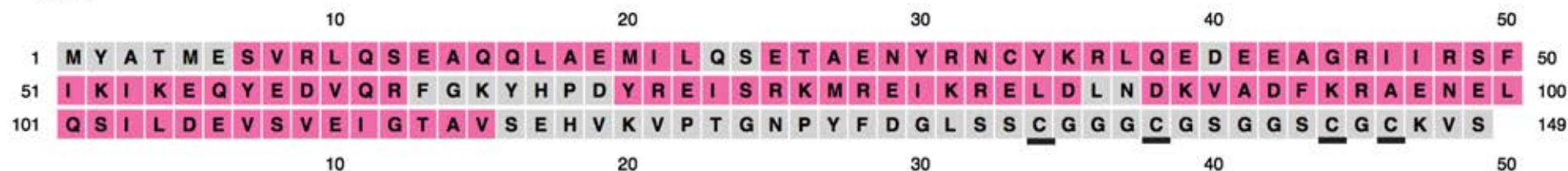

## RicT

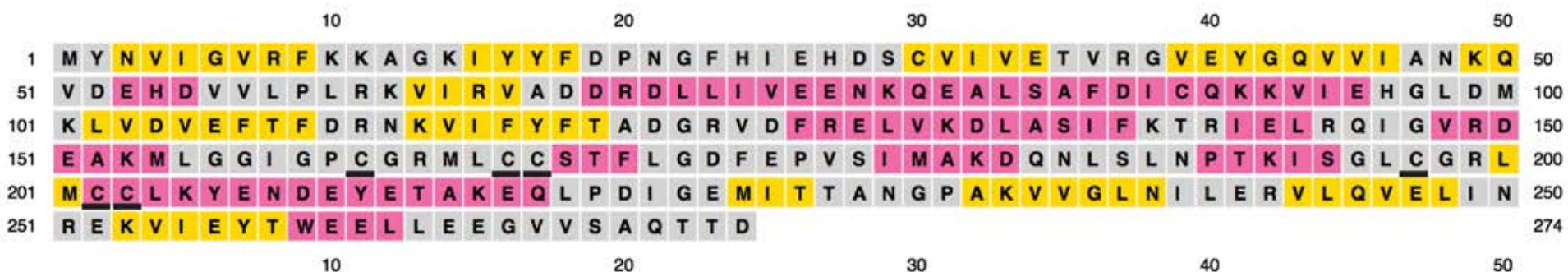

Strand

Helix

Coil

Supplement: FIG S6 [file mBio.01841-19-sf006.pdf]
