# Supplementary material for: Structure-Function Studies of the Bacillus subtilis Ric Proteins Identify the Fe-S Cluster-Ligating Residues and Their Roles in Development and RNA Processing
Source: mBio. 2019 Sep 17;10(5):e01841-19. doi: 10.1128/mBio.01841-19 (PMC6751060; doi:10.1128/mBio.01841-19)
Supplement: TABLE S1 [file mBio.01841-19-st001.pdf]

Table S1

Identities and similarities of Ric proteins[a]

Protein	%Identity/Similarity	
	Bsu vs *Gst*	*Bsu vs Ban*
RicT	78/88	77/90
RicA	63/87	66/83
RicF	56/80	59/76

[a] *Bsu: Bacillus subtilis*, *Gst*: *Geobacillus stearothermophilus*, *Ban*: *Bacillus anthracis*.
