# Supplementary material for: Structure-Function Studies of the Bacillus subtilis Ric Proteins Identify the Fe-S Cluster-Ligating Residues and Their Roles in Development and RNA Processing
Source: mBio. 2019 Sep 17;10(5):e01841-19. doi: 10.1128/mBio.01841-19 (PMC6751060; doi:10.1128/mBio.01841-19)
Supplement: FIG S5 [file mBio.01841-19-sf005.pdf]

A RicT

Bsu MYNVIGVRFK KAGKIYYFDP NGFHIEHDSC VIVETVRGVE YGQVVIANKQ VDEHDVVLPL 60
Gst MYTVVGVRFK KAGKIYYFDP GDFIIPVGEF VIVETARGIE YGKVVIANKQ VDENDVVLPL
Ban MYDVVGVRFK KAGKVYYFDP NQFDISENEF VIVETVRGIE YGKVVITKKQ VDENDVVLPL

Bsu RKVIRVADDR DLLIVEENKQ EALSAFDICQ KKVIEHGLDM KLVDVEFTFD RNKVIFYFTA 120
Gst KKVIRVANEK DKWVVEENKK AAREAYDICL RKVEEHGLEM KLVDVEYTFD RNKVIFYFTA
Ban KKVIRIANEN DRTIVEENKH AAKEAYQVCQ QKVVEHNLDM KLVDVEYTFD RNKIIFYFTA

Bsu DGRVDFRELV KDLASIFKTR IELRQIGVRD EAKMLGGIGP **C**GRML**CC**STF LGDFEPVSIK 180
Gst DGRVDFRELV KDLASIFRTR IELRQIGVRD EAKMLGGIGP **C**GRML**CC**STF LGDFEPVSIK
Ban DGRIDFRELV KDLAAIFRTR IELRQIGVRD EAKMLGGIGP **C**GRML**CC**STF LGDFEPVSIK

Bsu MAKDQNLSLN PTKISGL**C**GR LM**CC**LKYEND EYETAKEQLP DIGEMITTAN GPAKVVGLNI 240
Gst MAKDQNLSLN PTKISGL**C**GR LM**CC**LKYESE EYETAKEQLP DLGEYVETPY GFGKVVGLNI
Ban MAKDQNLSLN PAKISGL**C**GR LM**CC**LKYEND EYEAAKEQLP DLDQRIQTPH GTGRVIGLNI

Bsu LERVLQVELI NREKVIEYTW EELLEEGVVS AQTTD 275
Gst LERVLQIELP EHGRVVEYTL DELMKNGTLS IRVAD
Ban LERLIQVELV DKERIVEYTL DELVNKGVVS SQTTD

B …RicT

Bsu 151 …EAKMLGGIGP**C**GRML**CC**STFLGDFEPVSIKMAKDQNLSLNPTKISGL**C**GRLM**CC**LKYEND… 210
Gst …EAKMLGGIGP**C**GRML**CC**STFLGDFEPVSIKMAKDQNLSLNPTKISGL**C**GRLM**CC**LKYESE…
Ban …EAKMLGGIGP**C**GRML**CC**STFLGDFEPVSIKMAKDQNLSLNPAKISGL**C**GRLM**CC**LKYEND…
Lmo …EAKLLGGIGP**C**GRML**CC**STFLGDFEPVSIKMAKDQNLSLNPTKISGL**C**GRLM**CC**LKYEND…
Sau …EAKLLGGIGP**C**GRSL**CC**STFLGDFEPVSIKMAKDQNLSLNPTKISGA**C**GRLM**CC**LKYEND…
Lla …EAKMLGGIGP**C**GRML**CC**STFLGDFEPVSIKMAKDQNLSLNPTKISGL**C**GRLM**CC**LKYEND…
Spy …QARISGGLGP**C**GRPL**CC**SSFLGEFPKLSIKMAKNQNLSLSSGKSSGY**C**GHLL**CC**LQYEDQ…
Hmo …EAKMIGGIGC**C**GRVL**CC**ASFLGDFEPVSIRMAKDQNLSLNPTKISGI**C**GRLM**CC**LKFEND…
Mce …EAKMIGGIGC**C**GRPL**CC**ASFLGDFKPVSIRMAKGQGMSLNPTKISGI**C**GRLM**CC**LRYENS…
Cdi …EAKSIGGLGP**C**GRKL**CC**SSWLGDFQPVSIKMAKDQSLSLNPTKISGI**C**GRLF**CC**LKYEHD…
CXXXXCC------------X_{30}----------------CXXXXCC

C …RicF

Bsu 121 …VPTGNPYFDGLSS**C**GGG**C**GSGGS**C**G**C**KVS-COOH 149
Gst …KVPTGNPYFLSAG**C**SGG**C**RSGGG**C**G**C**RA-COOH
Ban …KVPTGNPFFDAGG**C**GGG**C**GTGGG**C**G**C**KKTG-COOH
C C C C

D …RicA

Bsu 121 …TGGDLLKGETGSKVKHSNNS**C**SL-COOH 143
Gst …TGGDVLRGETGAALRYNKHGG**C**H-COOH
Ban …TNGVDLKGETGAAVESKKGN**C**G**C**-COOH
