# Supplementary material for: Structure-Function Studies of the Bacillus subtilis Ric Proteins Identify the Fe-S Cluster-Ligating Residues and Their Roles in Development and RNA Processing
Source: mBio. 2019 Sep 17;10(5):e01841-19. doi: 10.1128/mBio.01841-19 (PMC6751060; doi:10.1128/mBio.01841-19)
Supplement: TABLE S2 [file mBio.01841-19-st002.pdf]

Table S2

Cloning primers[a]

	**For cloning RicT insert into pMiniMad2**
	F: GCTCGTGGATCCTCTTTATTCAAGATCAATGGATGCCCT<br>R: GCTCGTGGTACCATCCTTCTTGATACAGACGTGCC
	**For cloning RicA insert into pMiniMad2**
	F: AGGATCCCCGGGTACCAGGCACTCCGGCTGCTAAGAT<br>R: ATTCGAGCTCGGTACCTCCTCGTCATCCTCTTCCCAGTC
	**For cloning RicF insert into pMiniMad2**
	F: AGGATCCCCGGGTACCTCAGAGAACAGCCGATATGGTAC<br>R: ATTCGAGCTCGGTACCATCACCTGCCTGTTTTCTCTTATATGTAAA
	**For cloning *Ban* RicT in pQlink G**
	F: GGGGCCCCTGGGATCCTATGATGTAGTAGGTGTTCG<br>R: GGGTCCTAGGCGGCCGCTTAATCTGTGGTTTGACTCG
	**For cloning *Ban* RicF in pQlink N**
	F: ATTAACTATGGGATCCATGTGGTATGATGAATACTCG<br>R: GGGTCCTAGGCGGCCGCTTAAACTTTGATGGAAGAGGA
	**For cloning *Ban* RicA in pQlink N**
	F: ATTAACTATGGGATCCATGAAAGTATATTCGAAAGATG
	R: GGGTCCTAGGCGGCCGCTTAACAACCACAATTTCCTTTT
	**For cloning *Gst* RicT in pQlink G**
	F: GGGGCCCCTGGGATCCTTGTATACTGTCGTCGGTGTCC
	R: GGGTCCTAGGCGGCCGCTTAATCTGCGACACGAATGGACA
	**For cloning *Gst* RicF in pQlink N**
	F: ATTAACTATGGGATCCGTGAGGATCGCTACTCTCGAGCG
	R: GGGTCCTAGGCGGCCGCCTATGCTCGGCAGCCGCAG
	**For cloning *Gst* RicA in pQlink N**
	F: ATTAACTATGGGATCCATGGCGAAATATACGCGGGAT<br>R: GGGTCCTAGGCGGCCGCTTAATGGCAGCCGTCGTGC

[a]Relevant restriction sites are underlined.
