# Supplementary material for: Structure-Function Studies of the Bacillus subtilis Ric Proteins Identify the Fe-S Cluster-Ligating Residues and Their Roles in Development and RNA Processing
Source: mBio. 2019 Sep 17;10(5):e01841-19. doi: 10.1128/mBio.01841-19 (PMC6751060; doi:10.1128/mBio.01841-19)
Supplement: TABLE S3 [file mBio.01841-19-st003.pdf]

Table S3

Mutagenic primers [a]

Mutation	Mutagenic primers for expression of RicAFT in *Eco*
RicF C134S (*Ban*)	F: TTTTTGATGCTGGTGGC**TCT**GGTGGCGGTTGT<br>R:ATGGATTTCCTGTTGGAACTTTGATGGAAGAGGACAC
RicF C138S (*Ban*)	F:TGGCTGTGGTGGCGGT**TCT**GGTACTGGCGGAGGTTG<br>R:CCAGCATCAAAAAATGGATTTCCTGTTGGAACTTTGATGGA
RicF C144S (*Ban*)	F:GTACTGGCGGAGGT**TCT**GGTTGTAAAAAAACGGG<br>R:CACAACCGCCACCACAGCCACCAG
RicF C146S (*Ban*)	F: GTACTGGCGGAGGTTGTGGT**TCT**AAAAAAACGGG<br>R: CACAACCGCCACCACAGCCACCAG
RicF C134A (*Ban*)	F: TTTTTGATGCTGGTGGC**GCT**GGTGGCGGTTGT<br>R: ATGGATTTCCTGTTGGAACTTTGATGGAAGAGGACAC
RicF C138A (*Ban*)	F: TGGCTGTGGTGGCGGT**GCT**GGTACTGGCGGAGGTTG<br>R:CCAGCATCAAAAAATGGATTTCCTGTTGGAACTTTGATGGA
RicF C144A (*Ban*)	F: GTACTGGCGGAGGT**GCT**GGTTGTAAAAAAACGGG<br>R: CACAACCGCCACCACAGCCACCAG
RicF C146A (*Ban*)	F: GTACTGGCGGAGGTTGTGGT**GCT**AAAAAAACGGG<br>R: CACAACCGCCACCACAGCCACCAG
RicA C141S C143S (*Ban*)	F: AGTAAAAAAGGAAAT**TCT**GGT**TCT**TAAGCGGCCGCCTAGG<br>R: TTCTACCGCTGCGCCCGTTTCACCCTTCAATACATC
RicT C167S (*Ban*)	F: GTGGTCGTATGCTTTGT**TCT**TCTACTTTTTTAGGAGA<br>R: ATGGACCAATACCACCAAGCATCTTCGCTT
RicT C204S (*Ban*)	F: GGACGTCTCATGTGC**TCC**TTAAAATATGAGAGC<br>R: GCACAACCCGGAAATTTTCGTCGGGTTGAGC
RicT C161S (*Ban*)	F: GCGGCATTGGACCG**TCC**GGCCGCATGCTTT<br>R: CAAGCATTTTCGCCTCATCGCGCACCC
	**Mutagenic primers for expression of RicT in *Eco***
RicT C161S (*Gst*)	F: GGCGGCATTGGACCG**AGC**GGCCGCATGCTTTGC<br>R:AAGCATTTTCGCCTCATCGCGCACCCCGATTTGCCGCAG
RicT C166S (*Gst*)	F:TGCGGCCGCATGCTT**AGC**TGTTCGACCTTTCTT<br>R:CGGTCCAATGCCGCCAAGCATTTTCGCCTCA
RicT C167S (*Gst*)	F: GGCCGCATGCTTTGC**AGT**TCGACCTTTCTTGGC<br>R:GCACGGTCCAATGCCGCCAAGCATTTTCGCCTCA
RicT C198S (*Gst*)	F:AAAATTTCCGGGTTG**AGC**GGACGTCTCATGTG<br>R:CGTCGGGTTGAGCGATAAGTTTTGATCCTTCGCCATCTTGAT
RicT C203S (*Gst*)	F:TGCGGACGTCTCATG**AGC**TGCTTAAAATATGAG<br>R:CAACCCGGAAATTTTCGTCGGGTTGAG
RicT C204S (*Gst*)	F:GGACGTCTCATGTGC**AGC**TTAAAATATGAGAGC<br>R:GCACAACCCGGAAATTTTCGTCGGGTT
	**Mutagenic primers for expression in *Bsu* using pMiniMad2** [b]
RicT C161S (*Bsu*)	F: GGAGGAATCGGTCCT**TCC**GGAAGAATGCTTTGC
RicT C166S (*Bsu*)	F: GCGGAAGAATGCTT**TCC**TGTTCAACGTTTCT

RicT C167S (*Bsu*)	F: GAAGAATGCTT**TGC**TCTTCAACGTTTCTTGG
RicT C198S (*Bsu*)	F: AGATTTCGGGTCTT**TCC**GGACGATTGATGTG
RicT C203S (*Bsu*)	F: GCGGACGATTGATG**TCC**TGTCTAAAATATG
RicT C204S (*Bsu*)	F: GACGATTGATGTGC**TCT**CTAAAATATGAGAA
RicT C30S (*Bsu*)	F: ATAGAACATGACAGC**TCC**GTAATTGTAGAAACT
RicT C89S (*Bsu*)	F: TCAGCATTTGATATC**TCC**CAAAAGAAAGTGATT
RicA C141S (*Bsu*)	F: CATTCAAATAACAGC**TCT**TCTCTCTAAACACGG
RicF C134S (*Bsu*)	F: GACGGTTTGTCTTCA**TCC**GGAGGCGGCTGCGGT
RicF C138S (*Bsu*)	F: TCATGCGGAGGCGGC**TCC**GGTTCAGGCGGAAGC
RicF C144S (*Bsu*)	F: GGTTCAGGCGGAAGC**TCC**GGATGTAAAGTGTCC
RicF C146S (*Bsu*)	F: GGCGGAAGCTGCGGA**TCT**AAAGTGTCCTGACGA
RicF C33S (*Bsu*)	F: GAGAACTACCGCAAT**TCT**TACAAGCGTCTCCAG

[a]Mutations are indicated in boldface. When two primers are listed, a Phusion Site Directed Mutagenesis kit was used. When a single primer is listed the mutations were introduced using a Change-It kit.
