# Supplementary material for: Structure-Function Studies of the Bacillus subtilis Ric Proteins Identify the Fe-S Cluster-Ligating Residues and Their Roles in Development and RNA Processing
Source: mBio. 2019 Sep 17;10(5):e01841-19. doi: 10.1128/mBio.01841-19 (PMC6751060; doi:10.1128/mBio.01841-19)
Supplement: TABLE S4 [file mBio.01841-19-st004.pdf]

Table S4

Data collection and refinement statistics

	**Native**	**Native**
	RicA	RicA:RicF
**Data collection**		
Space group	C121	P62
Cell dimensions		
a, b, c (Å)	109.49, 52.21, 27.10	86.09, 86.09,87.64
α, β, γ (°)	90.00, 95.83, 90.00	90.00, 90.00, 120.00
Resolution (Å)	29.83-2.08 (2.12-2.08)	50.00-3.20(3.26-3.20)
Wavelength (Å)	1.18076	0.88557
Completeness (%)	99.8 (99.1)	99.9 (99.7)
R_{sym} (%)	14.1 (39.6)	8.0 (89.4)
R_{meas} (%)	15.1 (42.8)	8.5 (94.2)
Average I / σI	15.06 (5.04)	17.24 (2.24)
Redundancy	7.46	9.95
Total reflections	68,660	61,658
Unique reflections	9,208	6,195
$CC_{1/2}$	0.99 (0.93)	0.99 (.81)
**Refinement**		
R_{work} / R_{free} (%)	18.49/22.41	20.52/27.83
Number of non-hydrogen atoms	1032	1905
Protein	979	1905
Ions	1	0
Water	52	0
Wilson B factor (Å^2)	29.6	105.49
Average B-factor (Å^2)	36.80	144.79
RMS bond lengths (Å)	0.010	0.010
RMS bond angles (°)	1.588	1.250
Ramachandran statistics		
Favored (%)	99.17	96.05
Outliers (%)	0.00	0.88
PDB Identifier	6PRH	6PRK

Table S4. Data collection and refinement statistics. $R_{sym} = \Sigma_h \Sigma_i \mid I_i(h) - \langle I(h) \rangle \mid / \Sigma_h \Sigma_i I_i(h)$, where $I_i(h)$ is the ith measurement of h and $\langle I(h) \rangle$ is the mean of all measurements of I(h) for reflection h. $R_{work} = \Sigma \, ||F_o| - |F_c|| \, / \, \Sigma \, |F_o|$, calculated with a working set of reflections. R_{free} is R_{work} calculated with only the test set of reflections. Data for the highest resolution shell are given in parentheses. The structures were determined using single crystals.
